# Transcriptome Sequencing and *De Novo* Analysis of Cytoplasmic Male Sterility and Maintenance in JA-CMS Cotton

**DOI:** 10.1371/journal.pone.0112320

**Published:** 2014-11-05

**Authors:** Peng Yang, Jinfeng Han, Jinling Huang

**Affiliations:** 1 Department of Agronomy, Henan Agricultural University, Zhengzhou, Henan, China; 2 Department of Rural Development, Shanxi Agricultural University, Taigu, Shanxi, China; 3 Department of Agronomy, Shanxi Agricultural University, Taigu, Shanxi, China; New Mexico State University, United States of America

## Abstract

Cytoplasmic male sterility (CMS) is the failure to produce functional pollen, which is inherited maternally. And it is known that anther development is modulated through complicated interactions between nuclear and mitochondrial genes in sporophytic and gametophytic tissues. However, an unbiased transcriptome sequencing analysis of CMS in cotton is currently lacking in the literature. This study compared differentially expressed (DE) genes of floral buds at the sporogenous cells stage (SS) and microsporocyte stage (MS) (the two most important stages for pollen abortion in JA-CMS) between JA-CMS and its fertile maintainer line JB cotton plants, using the Illumina HiSeq 2000 sequencing platform. A total of 709 (1.8%) DE genes including 293 up-regulated and 416 down-regulated genes were identified in JA-CMS line comparing with its maintainer line at the SS stage, and 644 (1.6%) DE genes with 263 up-regulated and 381 down-regulated genes were detected at the MS stage. By comparing the two stages in the same material, there were 8 up-regulated and 9 down-regulated DE genes in JA-CMS line and 29 up-regulated and 9 down-regulated DE genes in JB maintainer line at the MS stage. Quantitative RT-PCR was used to validate 7 randomly selected DE genes. Bioinformatics analysis revealed that genes involved in reduction-oxidation reactions and alpha-linolenic acid metabolism were down-regulated, while genes pertaining to photosynthesis and flavonoid biosynthesis were up-regulated in JA-CMS floral buds compared with their JB counterparts at the SS and/or MS stages. All these four biological processes play important roles in reactive oxygen species (ROS) homeostasis, which may be an important factor contributing to the sterile trait of JA-CMS. Further experiments are warranted to elucidate molecular mechanisms of these genes that lead to CMS.

## Introduction

Cytoplasmic male sterility (CMS) is a maternally inherited trait in higher plants incapable of producing functional pollen [1]. However, CMS plants exhibit normal vegetative growth and female fertility, and this trait can be restored by nuclear genes known as *restorer-of-fertility* (*Rf*) genes [2]. On one hand, it has been widely accepted that CMS is closely related to mitochondrial genome rearrangement, which creates chimeric genes that disturb normal pollen development [3]. More than 50 mitochondrial genes have been identified as CMS-related in various plants [4]–[7], which are valuable in producing F1 hybrid cultivars with heterosis. On the other hand, changes in mitochondrial function trigger altered nuclear gene expression by a mysterious process called mitochondrial retrograde regulation (MRR) [8]. Much is still unknown about signalling pathways involved in this process, MRR targets, and how cells switch to programmed cell death (PCD) mode in the case of CMS.

Transcriptomic analysis using DNA microarray or RNA-seq technology has been implemented to investigate molecular markers or differentially expressed (DE) genes implicated in a variety of traits. Suzuki et al. [9] presented the first genome-wide transcriptome analysis of CMS and restoration in cotton using Affymetrix GeneChips Cotton Genome Array. They compared DE genes of floral buds at the meiosis stage between CMS-D8 and its restorer line, and found 458 (1.9%) DE genes including 127 up-regulated and 331 down-regulated ones. The most frequent DE gene group was involved in cell wall expansion [9].

In recent years, the emergence and advancement of next generation sequencing technology has made a breakthrough in life sciences, by offering unprecedented speed and cost efficiency to study genomic and transcriptomic data [10], [11]. Comparing with DNA microarray, RNA-seq has very low, if any, background signal, and has also been shown to be highly accurate for quantifying expression levels [12]. Researchers have used RNA-seq to study CMS in sweet orange (*Citrus sinensis*) [13], rape (*Brassica napus*) [14], and chili pepper (*Capsicum annuum* L.) [15], to give a few examples.

Zheng et al. [13] compared nuclear gene expression profiles of male sterile cybrid and fertile pummelo floral buds by RNA-seq analysis. *APETALA3* and *PISTILLATA* transcripts, which encode key transcription factors for stamen identification and are restricted to normal floral whorls, were repressed in the sterile line. As the authors stated, these citrus class-B MADS-box genes are likely to be targets for CMS retrograde signalling [13]. In *Brassica napus*, 3,231 genes of *Brassica rapa* and 3,371 genes of *Brassica oleracea* were detected with significantly different expression levels from young floral buds of sterile and fertile plants, which were derived from self-pollinated offspring of the F_1_ hybrid from novel restorer line NR1 and *Nsa* CMS line [14]. Altered expressions of genes involved in carbon metabolism, tricarboxylic acid cycle (TCA cycle), oxidative phosphorylation, oxidoreductase activity and pentatricopeptide repeat (PPR) proteins were observed by Yan et al. [14]. Liu et al. [15]’s study profiled anther transcriptomes of a chili pepper CMS line 121A and its restorer line 121C, and found the top three pathways covering the most DE unigenes were starch and sucrose metabolism, oxidative phosphorylation and plant-pathogen interaction [15].

However, unbiased transcriptome sequencing analysis of CMS in cotton is lacking. To narrow this gap, we isolated, sequenced and quantified transcriptomes of floral buds at the sporogenous cells stage (SS) and microsporocyte stage (MS), which were demonstrated to be the two most important stages for pollen sterility [16] in JA-CMS, a CMS line cultivated by Cotton Breeding Lab of Shanxi Agricultural University (Please see materials and methods for details about JA-CMS). The obtained transcriptomic sequences were then assembled, annotated, and analysed to discover DE genes between the two lines at the two specific stages, to find pathways that were enriched with these DE genes, and to further determine potential key genes functional in CMS.

## Materials and Methods

### Plant materials and RNA extraction

JA-CMS and its maintainer line JB cotton plants were grown in the fields of Shanxi Agricultural University, Taigu, Shanxi, China. The two lines were isolated by repeated backcrossing of (*Gossypium hirsutum*×*G. thurberi*)×(*G. arboreum*×*G. hirsutum*) for more than 10 generations using MB177, one kind of *G. hirsutum*, as the recurrent parent. With 100% sterile plants, JA-CMS is unique in the following three ways: it has the genetic background of both *G. thurberi* and *G. arboreum*; it matures around 20 days earlier than 104-7A [17], another CMS line cultivated by Chinese scientists through backcrossing of *G. hirsutum* and *G. barbadense*; it is conditioned by *G. hirsutum* cytoplasm, which is different from CMS lines of *G. harknessii*, 104-7A and *G. trilobum*, CMS-D8, based on origins and phenotypes. Please refer to the supplementary note for a detailed comparison of JA-CMS, 104-7A, CMS-D2 and CMS-D8. Our group has studied morphological and cellular characteristics of JA-CMS, determined major development stages for pollen abortion, and conducted biochemical and molecular biological research on CMS. In collaboration with the Institute of Cotton Study, Shanxi Agricultural Academy, a restorer line of JA-CMS named JB was also cultivated [16], [18].

Because of the stable correlation between development stages of pollen mother cells and floral bud morphology [19], following the method described by Zhao and Huang [18], three floral buds, with diameters less than 2 mm or between 2 and 3 mm, were collected from five sterile and fertile plants at the same time, respectively, to confirm the correlation in our samples using cytological methods. As the results suggested, for JA-CMS and its maintainer line, a floral bud is at the SS stage when its diameter is between 1.5–2.2 mm, and at the MS stage when its diameter reaches 2.2–2.6 mm.

Floral buds were harvested from at least 50 plants of each line at apporoximately the same time of the day; 2 g of floral buds were mixed as a pool based on different stages and cotton lines. Pooled materials were snap-frozen in liquid nitrogen and stored at −80°C for RNA preparation. Total RNA was isolated using an RN37-EASYspin RNA extraction kit (Aidlab Biotechnologies, China) according to the manufacturer’s protocol. The integrity of total RNA was checked by 1% agarose gel electrophoresis; the concentration and purity were determined using NanoDrop (Thermo Scientific, USA) and Agilent 2100 Bioanalyzer (Agilent, USA). A total amount of 3 µg RNA per sample was used as input material for the RNA sample preparations. All four samples had RIN (RNA Integrity Number) values above 8.

### Library preparation and sequencing

Sequencing libraries were generated using Illumina TruSeq RNA Sample Preparation Kit (Illumina, USA) following manufacturer’s recommendations and four index codes were added to attribute sequences to each sample. Briefly, after integrity, concentration and purity check, poly-(T) oligo-attached magnetic beads were utilized for mRNA enrichment. Then fragmentation buffer was added to break mRNA into short fragments, which served as templates for the first strand cDNA synthesis with random hexamer-primer. Buffer, dNTPs, RNase H and DNA polymerase I were added afterwards to synthesize the second strand. Remaining overhangs were converted into blunt ends via exonuclease/polymerase activities and enzymes were removed. The double-stranded cDNA was purified with AMPure XP beads, followed by end repair, poly-(A) addition and sequencing adaptor ligation. Fragments preferentially 200 bp in length were enriched using Illumina PCR Primer Cocktail in a 10 cycle PCR amplification to obtain the cDNA library. To ensure quality of the library, Qubit 2.0 (Life Technologies, USA), Agilent 2100 (Agilent, USA) and quantitative PCR were used for initial quantification, insert size check and effective concentration determination (library effective concentration>2 nM), respectively.

The clustering of the index-coded samples was performed on a cBot Cluster Generation System using TruSeq PE Cluster Kit v3-cBot-HS (Illumina, USA) according to the manufacturer’s instructions. After cluster generation, the library was sequenced on Illumina HiSeq 2000 platform; 100 bp paired-end reads were generated. Low-quality reads containing ambiguous nucleotides or adaptor sequences were removed from the raw reads to gain clean reads, which were then assembled using Trinity to construct unique consensus sequences without a reference genome [20].

### Bioinformatics analysis

Seven databases were used for unigene annotation, including the NCBI protein database (http://www.ncbi.nlm.nih.gov/protein), the NCBI nucleotide database (http://www.ncbi.nlm.nih.gov/nucleotide), the Pfam database (http://pfam.xfam.org/), the euKaryotic Orthologous Groups (KOG; http://www.ncbi.nlm.nih.gov/COG/), the UniProt Knowledgebase (UniProtKB)/Swiss-Prot (http://www.ebi.ac.uk/uniprot), the KEGG Orthology database (KO; http://www.genome.jp/kegg/ko.html), and the Gene Ontology (GO; http://www.geneontology.org/). We performed these annotations by using a combination of BLAST (http://blast.ncbi.nlm.nih.gov/Blast.cgi), HMMER (http://hmmer.janelia.org/), Blast2GO (http://www.blast2go.com/b2ghome), and KEGG Automatic Annotation Server (KAAS; http://www.genome.jp/kegg/kaas/).

Using the transcriptome assembled by Trinity [20] as reference, clean reads of each sample were mapped to it by RSEM (RNA-Seq by Expectation-Maximization) [21]. For biologically non-duplicating samples, mapped read counts were normalized through applying trimmed mean of M values (TMM) method [22]. Then normalized counts were analysed and unigenes showing significant expression differences were determined using DEGseq [23], with FDR q-value threshold set to 0.005 and absolute value of expression fold change set to 2.

After selection of unigenes with significant transcript abundance difference (DE genes), we conducted enrichment analyses, i.e. GO and KEGG analysis, to elucidate distributions of these unigenes based on their functions and attached biological pathways. GO analysis was performed using Goseq [24], which is based on Wallenius non-central hypergeometric distribution. KEGG analysis utilizes hypergeometric test to search DE genes that are significantly enriched in certain KEGG pathways compared with other genes in the genome. KOBAS 2.0 [25] was used for KEGG analysis. GO categories and KEGG pathways with FDR q-value ≤ 0.05 were selected as significantly enriched.

KEGG analysis results are presented graphically using scatter plots, with three parameters for enrichment level assessment, which are rich factor, the ratio between counts of DE genes and all annotated genes enriched in a certain pathway (higher the ratio, more significant the pathway); qvalue, P value after multiple hypothesis testing correction with a range between 0 and 1 (closer to 0, more significant the pathway); and number of genes enriched. Twenty most significant pathways were plotted, when more than twenty pathways were identified for each of the four comparisons.

### Quantitative RT-PCR

Quantitative RT-PCR (qRT-PCR) was performed to validate transcript abundance of unigenes observed by RNA-seq. Primer-BLAST (http://www.ncbi.nlm.nih.gov/tools/primer-blast/) was used to design gene-specific primers for seven randomly selected DE genes plus elongation factor-1α gene as the internal control (Table S1). Reactions were carried out using the SYBR *Premix Ex Taq* II (Tli RnaseH Plus) Kit (Takara, USA) in the iQ5 Multicolour Real-Time PCR Detection System (Bio-Rad, USA), according to the manufacturer’s instructions.

The first-strand cDNA was synthesized using the PrimeScript RT Master Mix Kit (Takara, USA) under the following conditions: 37°C for 15 min (reverse transcription) and 85°C for 5 s (denaturing reverse transcriptase). Then amplification reactions of 20 µL volume consisting of 10 µL SYBR *Premix Ex Taq* II, 0.8 µL of 10 µM forward and reverse primers each, 1 µL cDNA template and sterile water, were conducted, with cycling parameters as follows: 95°C for 30 s, 40 cycles of 95°C for 5 s and 60°C for 30 s. Relative expression levels of unigenes from different samples were calculated using 2^−ΔΔCT^ method [26]. The qRT-PCR was conducted with 3 replicates for each sample, and data were indicated as means ± standard errors (n = 3) in Figure S1.

### Data access

The transcriptome sequencing data from this study have been deposited in the NCBI SRA database and are accessible through accession numbers SRX547770, SRX547777, SRX547779, and SRX547781 (http://www.ncbi.nlm.nih.gov/sra).

## Results

### RNA sequencing and gene annotations

A total of 2.8×10^8^ raw reads were obtained from the Illumina sequencing platform. Each stage of either sterile or fertile line had around 6.8 ± 0.6 giga base pairs (Gb) of sequencing data. After data quality control as described in materials and methods, 97.54% ± 0.26% of the raw reads were clean reads for each of the four samples (Table 1). Trinity [20] reconstructed all the clean reads into a *de novo* reference transcriptome; the longest transcript for each gene was recognized as the unigene. Following these steps, 206,496 transcripts and 86,093 unigenes were identified, length distributions of which are presented in Figure S2 and S3. The transcripts and unigenes are between 201 bp and 17,233 bp in length; the mean lengths of the transcripts and unigenes are 1,184 bp and 755 bp, respectively. Around 36.3% of the transcripts and 59.8% of the unigenes are within the range of 200–500 bp.

**Table 1:** Transcriptome sequencing data quality.

Sample	Raw reads	Clean reads	Clean bases	Error (%)	Q20 (%)	Q30 (%)	GC (%)
JA-CMS at the SS stage	66695222	64995444	6.50 Gb	0.03	98.05	93.60	43.79
JA-CMS at the MS stage	77998708	75844362	7.58 Gb	0.03	98.02	93.57	43.65
JB at the SS stage	71264156	69566718	6.96 Gb	0.03	98.01	93.45	43.74
JB at the MS stage	62779022	61431768	6.14 Gb	0.03	98.11	93.71	43.75

Notes: Gb = Giga base pair; Q20 represents an error rate of 1 in 100, with a corresponding call accuracy of 99%; Q30 represents an error rate of 1 in 1000, with a corresponding call accuracy of 99.9%.

The percentage of successfully annotated unigenes differs among the seven databases specified in the Materials and Methods part, with the NCBI protein database having the highest, 42.05% and KO providing the lowest, 11.76%. Only 5.15% of the unigenes are annotated in all the databases, while 49.47% are annotated in at least one database. Annotations are provided whenever available.

### Genome-wide analysis of DE genes

**Figure 1:**
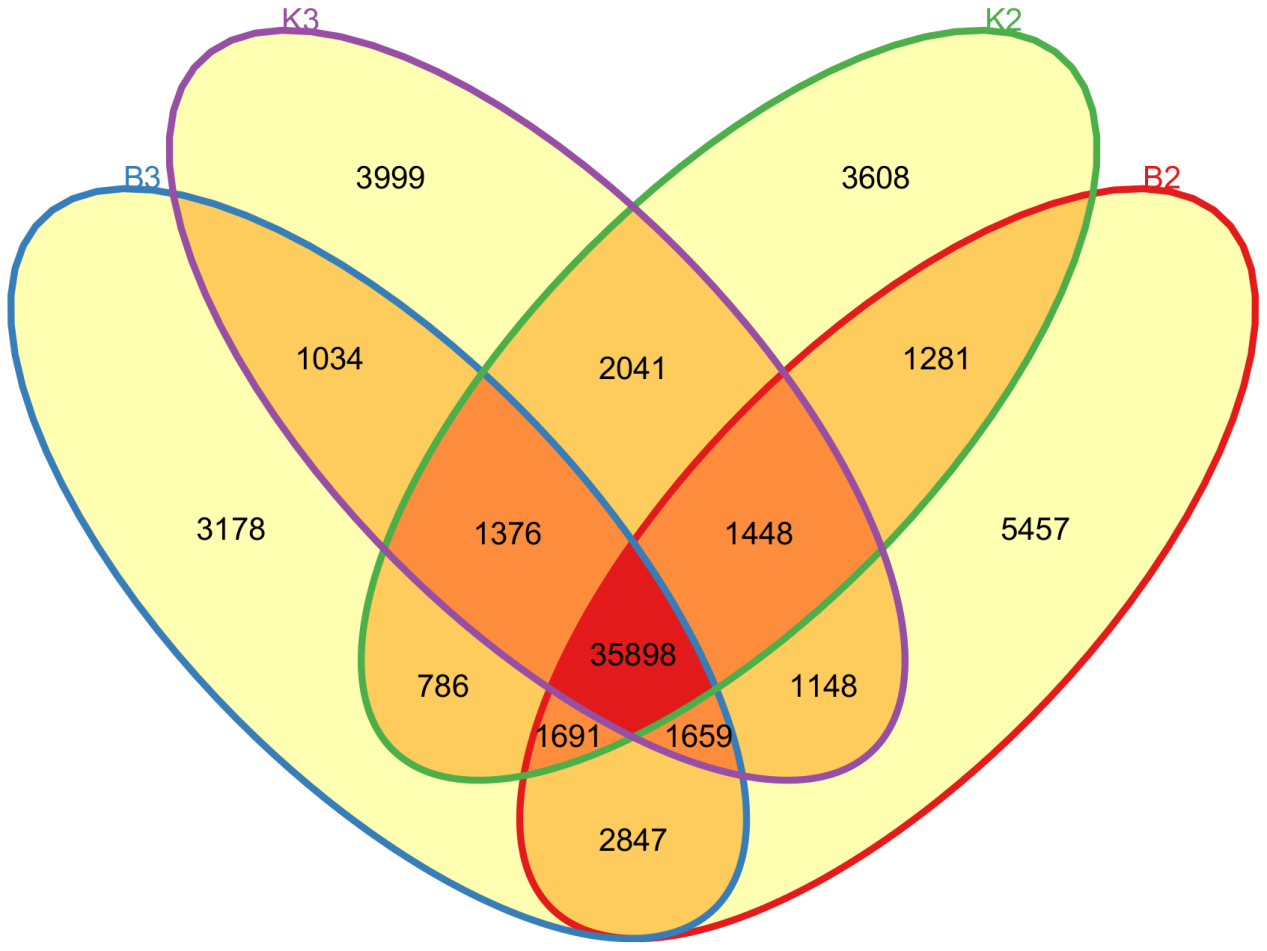
Venn diagram of DE gene counts at the SS and MS stages for JA-CMS and JB floral buds. B2 = SS stage of JA-CMS, K2 = SS stage of JB, K3 = MS stage of JB, B3 = MS stage of JA-CMS (from right to left).

Gene expression levels at the SS or MS stage for JA-CMS and JB floral buds were quantified and compared using RSEM [21], TMM [22], and DEGseq [23]. At the SS stage, there were 11,111 genes uniquely expressed in JA-CMS plants and 7,811 in JB plants; 40,318 genes were shared between the two lines at this stage. At the MS stage, 8,502 and 8,636 genes were specifically expressed in JA-CMS and JB lines, and the number of commonly expressed genes was 39,967. Regarding gene expression level comparison between the two stages of the same plant material, JA-CMS plants had 9,334 genes uniquely expressed at the SS stage and 6,374 at the MS stage; 42,905 genes were expressed at both stages; and there were 7,366 and 7,840 genes expressed at the SS and MS stages of JB plants, respectively, while 40,763 genes were commonly found at both stages (Figure 1).

Due to the large number of genes, we used FDR q value<0.005 combined with |log_2_(fold change)|>1 to select DE genes between the two materials at the same stage, or between different stages of the same material. By comparing with the JB line, JA-CMS plants had 709 genes with significantly different expression levels at the SS stage, among which 293 were up-regulated and 416 were down-regulated (Table S2). At the MS stage, 644 genes passed the significance threshold, with 263 up-regulated and 381 down-regulated in JA-CMS plants (Table S3). Fewer genes showed significant expression difference between the two stages of the same material. Seventeen genes, with 8 up-regulated and 9 down-regulated, had significantly different expression levels at the MS stage of JA-CMS comparing with the SS stage (Table S4). There were 38 DE genes showing significance in JB line between the two stages, among which 29 were up-regulated and 9 were down-regulated at the MS stage (Table S5).

All the 854 DE genes that showed significance in at least one of the four comparisons have 80.76 ± 158.10 and 78.07 ± 150.42 RPKM (Reads per kilobase per million) values at the SS and MS stages of JA-CMS, respectively. The RPKM values for JB plants have larger means and standard deviations than JA-CMS at both stages: 91.40 ± 301.55 (SS stage) and 96.25 ± 304.16 (MS stage). These values indicate reasonable coverage for the DE genes. Please refer to Figure S4 for the density distributions of log10(RPKM) for the four samples.

### Enrichment analysis of DE genes

Enrichment analysis was performed for the purpose of identifying major functions or pathways associated with CMS when genes function co-ordinately under certain biological mechanisms instead of individually to cause specific phenotype, which is the case for CMS. This type of analysis prioritizes DE genes for further investigation, if many DE genes are identified, and significantly increases statistical power to find predisposing genes, if few are available [27].

#### GO analysis

GO covers three domains: biological process (BP), cellular component (CC) and molecular function (MF). DE genes between the two materials at the SS stage were significantly enriched in 27 GO categories. Processes, components and functions related to reduction-oxidation (redox) reactions, photosynthesis, and transcription factors showed up in two out of the three GO domains. Oxidation-reduction process (GO:0055114) encompassed 100 DE genes with the smallest q value of all the 27 categories, which equals to 7.9×10^−4^ (Table 2). We also conducted GO analysis on up-regulated and down-regulated DE genes at the SS stage, separately (Figure S5 and S6). For the up-regulated genes, their product properties were mostly enriched in the biological process of photosynthesis and cellular components of photosynthetic membranes with both q values equal to 6.31×10^−6^; besides these two categories, other processes, components and functions related to photosynthesis were also prominent, such as photosystem (q = 7.48×10^−6^), thylakoid part and thylakoid (q = 1.50×10^−5^; Figure S5). On the other hand, down-regulated gene product properties were over-represented in only four categories, which are all pertinent to redox reactions (Figure S6).

**Table 2:** GO analysis results of DE genes at the SS and MS stages between JA-CMS and JB.

GOaccessionID	Description	Domain	SS stage			MS stage				
			Pvalue	Qvalue	DEgene #	Pvalue		Qvalue		DEgene #
GO:0055114	Oxidation-reduction process	BP	1.53E-07	7.90E-04	100	6.79E-08		2.43E-04		92
GO:0016705	Oxidoreductase activity, acting on paired donors, withincorporation or reduction of molecular oxygen	MF	6.46E-07	1.66E-03	35	1.02E-07		2.43E-04		34
GO:0001071	Nucleic acid binding transcription factor activity	MF	1.58E-06	2.04E-03	48	Notsignificant				
GO:0003700	Sequence-specific DNA binding transcription factor activity	MF	1.58E-06	2.04E-03	48	Notsignificant				
GO:0016491	Oxidoreductase activity	MF	2.73E-06	2.44E-03	96	4.37E-07		4.51E-04		89
GO:0005667	Transcription factor complex	CC	2.83E-06	2.44E-03	55	Notsignificant				
GO:0009521	Photosystem	CC	4.40E-06	3.24E-03	17	6.62E-05		3.10E-02		14
GO:0034357	Photosynthetic membrane	CC	5.60E-06	3.61E-03	18	6.58E-05		3.10E-02		15
GO:0046872	Metal ion binding	MF	8.76E-06	4.86E-03	116	1.89E-07		2.43E-04		110
GO:0043169	Cation binding	MF	9.43E-06	4.86E-03	118	1.83E-07		2.43E-04		112
GO:0005654	Nucleoplasm	CC	1.20E-05	5.13E-03	58	Notsignificant				
GO:0044451	Nucleoplasm part	CC	1.20E-05	5.13E-03	58	Notsignificant				
GO:0015979	Photosynthesis	BP	1.40E-05	5.54E-03	26	1.07E-04		4.59E-02	22	
GO:0008152	Metabolic process	BP	1.57E-05	5.77E-03	394	Notsignificant				
GO:0044436	Thylakoid part	CC	2.34E-05	8.05E-03	18	Notsignificant				
GO:0009579	Thylakoid	CC	2.81E-05	8.58E-03	18	Notsignificant				
GO:0016706	Oxidoreductase activity, acting on paired donors, with incorporationor reduction of molecular oxygen, 2-oxoglutarate as one donor,and incorporation of one atom each of oxygen into both donors	MF	2.83E-05	8.58E-03	14	7.40E-06		5.45E-03	14	
GO:0031981	Nuclear lumen	CC	7.38E-05	2.11E-02	63	Notsignificant				
GO:0032787	Monocarboxylic acid metabolic process	BP	8.31E-05	2.25E-02	24	Notsignificant				
GO:0009538	Photosystem I reaction center	CC	9.21E-05	2.37E-02	5	5.10E-05	2.92E-02		5	
GO:0044428	Nuclear part	CC	1.07E-04	2.62E-02	75	Notsignificant				
GO:0043233	Organelle lumen	CC	1.21E-04	2.71E-02	63	Notsignificant				
GO:0070013	Intracellular organelle lumen	CC	1.21E-04	2.71E-02	63	Notsignificant				
GO:0044710	Single-organism metabolic process	BP	1.47E-04	3.15E-02	184	Notsignificant				
GO:0031974	Membrane-enclosed lumen	CC	1.55E-04	3.19E-02	63	Notsignificant				
GO:0051213	Dioxygenase activity	MF	1.90E-04	3.77E-02	15	3.09E-06	2.65E-03		17	
GO:0048037	Cofactor binding	MF	2.17E-04	4.13E-02	38	Notsignificant				
GO:0046914	Transition metal ion binding	MF	Notsignificant			2.03E-05	1.31E-02		83	

Notes: 1) BP = Biological process, CC = Cellular component, MF = Molecular function; Q value = FDR corrected p value. 2) Only GO categories with FDR q value ≤ 0.05 are presented here. 3) GO categories are arranged based on q values from smallest to largest at the SS stage.

For the MS stage, product properties of DE genes between JA-CMS and JB lines were separated into 12 categories (Table 2). The most significant categories (q = 2.43×10^−4^) were oxidation-reduction process, oxidoreductase activity (acting on paired donors, with incorporation or reduction of molecular oxygen), cation binding and metal ion binding. Same as the SS stage, redox reactions and photosynthesis covered most of the GO categories. Additional GO analysis on up-regulated DE genes showed that, besides photosynthesis related categories, 7 out of the 43 significantly enriched classes were pertinent to transcription (Figure S7). Consistent with the SS stage analysis results, every category identified in GO analysis on down-regulated DE genes at the MS stage was involved in redox reactions (Figure S8). By comparing JA-CMS and JB lines at the two stages, it became obvious that genes implicated in redox reactions were persistently down-regulated, while genes related to photosynthesis were continuously up-regulated.

GO analysis did not find any significantly enriched category concerning up-regulated DE genes at the MS stage comparing with the SS stage of sterile plants; however, 24 categories were over-represented by down-regulated DE gene products, all of which were related to ATP biosynthesis (Figure S9). Combined analysis of up-regulated and down-regulated DE genes revealed the same category distribution as the down-regulated analysis. No GO enrichment was detected for DE genes between the two development stages of fertile plants.

#### KEGG analysis

**Figure 2:**
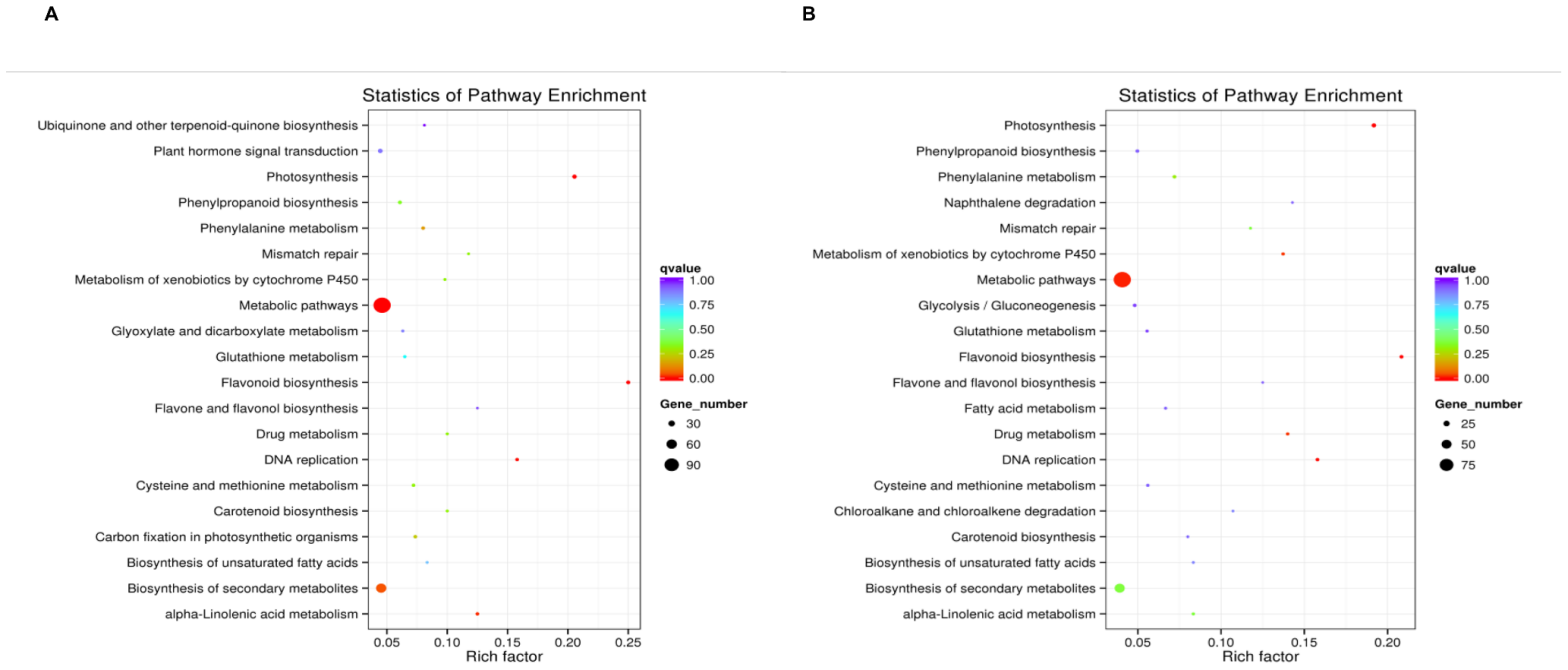
KEGG analysis results of DE genes at the SS and MS stages comparing JA-CMS and JB lines. Rich factor is the ratio between counts of DE genes and all annotated genes enriched in a certain pathway; qvalue is P value after multiple hypothesis testing correction with a range between 0 and 1. Twenty most significant pathways were plotted, when more than 20 pathways were identified. A) Results for the SS stage. B) Results for the MS stage.

There were 134 pathways determined for DE genes between JA-CMS and JB plants at the SS stage, among which the most significant ones were “photosynthesis” (gene number [N] = 15), “flavonoid biosynthesis” (N = 12), “metabolic pathways” (N = 112), “DNA replication” (N = 9), and “alpha-linolenic acid metabolism” (N = 9; Figure 2A). Consistent with GO analysis, “photosynthesis” was significantly enriched with up-regulated DE genes, as well as “flavonoid biosynthesis” and “DNA replication” (Figure S10). Down-regulated DE genes were over-represented in “alpha-linolenic acid metabolism” (Figure S11). Four out of the five pathways identified by KEGG for the MS stage were the same as the SS stage, which are “photosynthesis” (N = 14), “flavonoid biosynthesis” (N = 10), “DNA replication” (N = 9), and “metabolic pathways” (N = 99). Another pathway showing significance was “drug metabolism” (N = 7) among the total 140 pathways identified at the MS stage (Figure 2B). The three pathways significantly enriched with up-regulated genes at this stage were the same as the SS stage (Figure S12); “Metabolism of xenobiotics by cytochrome P450” and “drug metabolism” were enriched with down-regulated genes (Figure S13).

Because fewer DE genes were determined between the two stages of the same material, no significantly enriched pathway was detected.

### qRT-PCR verification on gene expression patterns

**Figure 3:**
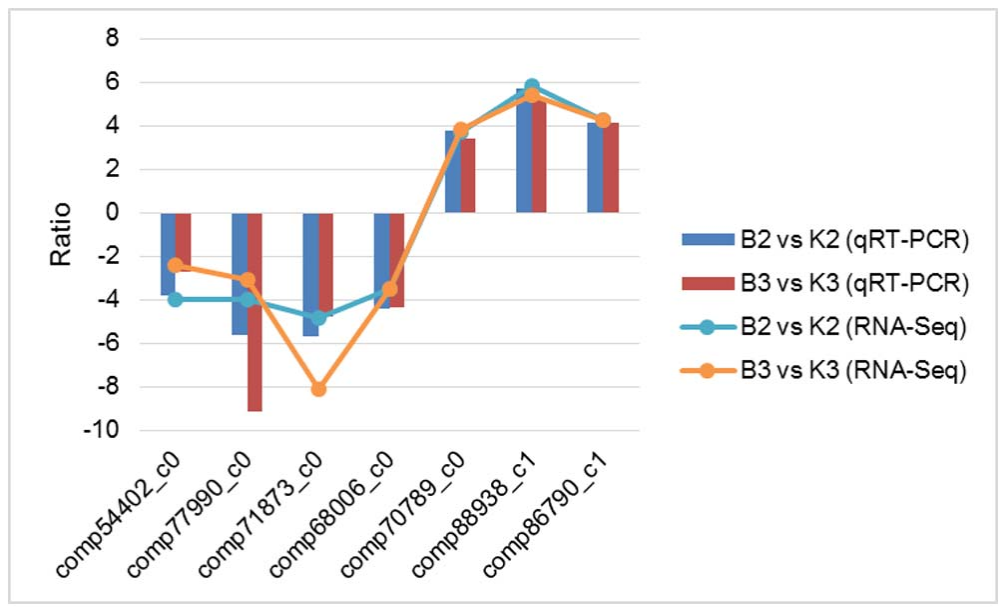
Quantitative RT-PCR validations for expression profiles of seven selected DE genes. Comp54402_c0 is 1-aminocyclopropane-1-carboxylate synthase gene, comp77990_c0 is alpha-expansin 11 precursor gene, comp71873_c0 is a predicted protein gene, comp68006_c0 is a conserved hypothetical protein gene, comp70789_c0 is chloroplast chlorophyll A–B binding protein gene, comp88938_c1 is transcription factor LHY gene, comp86790_c1 is *G. hirsutum* cab gene for chlorophyll A–B binding protein. Ratio stands for log2 ratio of relative expression levels for qRT-PCR and RPKM (Reads per kilobase per million) values for RNA sequencing, respectively. B2 = SS stage of JA-CMS, K2 = SS stage of JB, B3 = MS stage of JA-CMS, K3 = MS stage of JB.

Although quantitative differences existed between qRT-PCR analysis results and sequencing data for the selected seven DE genes (Table S1 and Figure S1), overall expression tendencies were the same (Figure 3). For example, comp54402_c0 is 1-Aminocyclopropane-1-carboxylic acid synthase (ACS) gene included in amino acid metabolism pathway, expression of which was down-regulated in sterile plants. Sequencing data showed 16-fold difference of transcript abundance between JA-CMS and JB lines at the SS stage, while qRT-PCR analysis yielded around 14-fold difference. Chlorophyll a/b-binding protein gene, comp70789_c0, was up-regulated in sterile plants compared with fertile counterparts at the MS stage based on sequencing data (15-fold), which was confirmed by the 11-fold increase shown in the corresponding qRT-PCR analysis result.

## Discussion

As a result of our unbiased transcriptome sequencing analysis of JA-CMS and its maintainer line, four candidate functional categories or pathways related to CMS have been identified, which include two down-regulated gene groups in JA-CMS: genes related to redox reactions and alpha-linolenic acid metabolism, and two up-regulated gene groups: genes pertaining to photosynthesis and flavonoid biosynthesis. Discussion of these genes would not be possible without mentioning the preceding study results of JA-CMS.

According to previous cytological studies, premature microspore abortion and tapetum degradation at the SS stage is one prominent characteristic of JA-CMS, and enzymatic activities of peroxidase, superoxide dismutase, cytochrome oxidase and succinate dehydrogenase were found significantly lower in JA-CMS comparing with its fertile counterpart [16]. Tapetum PCD plays an important role in pollen development [28]–[30], and abnormal reactive oxygen species (ROS) level is believed to be a signal promoting PCD in plants [31]. Disrupted ROS homeostasis was found in several CMS systems, e.g., excessive accumulation of O_2_^−^, H_2_O_2_ and malondialdehyde (MDA) existed in CMS cotton at the abortion peak [32], and mitochondria of a rice CMS line suffered from serious oxidative stress, which was induced by abnormal increased ROS at the meiosis stage [33].

In plants, chloroplasts are a major site of ROS generation, where photosynthetic electron transfer chains produce O_2_^−^ [34]. Because ROS can cause damage to proteins, lipids, and DNA, its production and removal must be strictly controlled [35]. Plants possess complex antioxidative defense system comprising of nonenzymatic and enzymatic components to scavenge ROS. Nonenzymatic components include the major cellular redox buffers ascorbate (AsA) and glutathione (γ-glutamyl-cysteinyl-glycine, GSH), which act as cofactors for enzymes of AsA-GSH cycle ascorbate peroxidase (APX), monodehydroascorbate reductase (MDHAR), dehydroascorbate reductase (DHAR), glutathione reductase (GR), glutathione peroxidase (GPX), and glutathione S transferase (GST) [36].

Based on both GO and KEGG analysis results, up-regulated genes in JA-CMS were significantly enriched in photosynthetic categories and pathways, while oxidation-reduction process and oxidoreductase activity categories were significantly over-represented with down-regulated DE genes at the SS and MS stages in sterile plants. There are 14 DE genes up-regulated in JA-CMS belonged to the KEGG pathway of “photosynthesis”, among which Photosystem I subunit PsaO encoding gene (comp65579_c0) showed the maximum fold change of expression at both SS (14-fold) and MS (12-fold) stages (Table 3).

**Table 3:** Up-regulated photosynthesis and flavonoid biosynthesis related genes in JA-CMS at the SS and/or MS stages.

Category	Function	Gene ID	SS stage			MS stage		
			Fold	P value	Q value	Fold	P value	Q value
Photosynthesis	Photosystem I subunit X	comp53760_c0	2.53	2.23E-08	2.32E-06	2.56	1.23E-07	1.23E-05
	Plastocyanin	comp65386_c0	3.02	1.48E-30	7.37E-28	2.61	6.19E-24	2.34E-21
	Photosystem I subunit PsaO	comp65579_c0	3.77	8.58E-17	1.99E-14	3.61	4.29E-14	8.67E-12
	Photosystem II PsbW protein	comp65639_c0	2.34	5.34E-07	4.77E-05	2.35	1.28E-06	1.14E-04
	Photosystem II 10kDa protein	comp68020_c0	1.17	3.48E-10	4.54E-08	Not significant		
	Photosystem II PsbY protein	comp68793_c0	1.72	4.38E-07	3.96E-05	Not significant		
	Photosystem I subunit XI	comp70643_c0	1.83	3.25E-15	6.79E-13	1.60	9.63E-11	1.44E-08
	Ferredoxin	comp71277_c0	3.11	4.21E-18	1.07E-15	2.68	9.12E-15	1.94E-12
	Photosystem I subunit IV	comp72189_c0	1.36	1.59E-08	1.70E-06	1.07	1.35E-05	1.01E-03
	Photosystem II oxygen-evolving enhancer protein 1	comp73151_c0	2.96	7.52E-57	9.24E-54	2.74	1.66E-45	1.70E-42
	Photosystem II 22kDa protein	comp73229_c0	2.28	2.04E-14	4.05E-12	2.14	4.45E-12	7.67E-10
	Photosystem II oxygen-evolving enhancer protein 2	comp73376_c0	1.18	2.53E-10	3.38E-08	Not significant		
	Photosystem I subunit III	comp73879_c0	1.83	2.89E-20	8.55E-18	1.65	1.73E-14	3.61E-12
	Photosystem II oxygen-evolving enhancer protein 3	comp84774_c2	1.83	4.26E-10	5.51E-08	1.55	2.13E-07	2.10E-05
Flavonoid biosynthesis	Chalcone synthase	comp72843_c0	2.23	2.61E-41	2.07E-38	1.63	1.96E-29	1.03E-26
		comp72843_c1	2.11	6.78E-13	1.18E-10	1.48	4.46E-09	5.49E-07
		comp84628_c1	2.06	5.90E-62	8.22E-59	1.56	2.09E-43	2.00E-40
		comp84628_c0	1.88	2.55E-78	5.51E-75	1.56	2.23E-68	4.13E-65
	Anthocyanidin reductase	comp73018_c0	1.27	1.48E-39	1.09E-36	Not significant		
	Leucoanthocyanidin dioxygenase	comp75804_c0	2.17	1.28E-94	4.02E-91	1.89	3.33E-81	8.49E-78
	Bifunctional dihydroflavonol 4-reductase/flavanone 4-reductase	comp75884_c0	1.75	2.37E-69	3.81E-66	1.54	8.80E-60	1.35E-56
	Trans-cinnamate 4-monooxygenase	comp80855_c0	1.47	1.42E-71	2.54E-68	1.09	7.25E-44	7.05E-41
	Flavonoid 3′,5′-hydroxylase	comp81439_c0	2.50	1.74E-93	4.97E-90	1.86	3.08E-57	4.39E-54
	Naringenin 3-dioxygenase	comp82651_c1	1.67	5.00E-94	1.49E-90	1.25	6.40E-67	1.12E-63

Note: Fold = log_2_(fold change). Significant DE genes were determined based on |log_2_(fold change)|>1 and FDR q value<0.005.

Down-regulated oxidoreductase genes in this study comprise 8 iron/ascorbate family oxidoreductase genes, 1 MDHAR gene, 1 GPX gene and 5 GST genes, which explains the significant enrichment of “drug metabolism” in KEGG analysis of down-regulated genes at the MS stage (Table 4). Within these genes, MDHAR gene had the maximum 17-fold decrease of its transcript abundance level in JA-CMS, which may be a major player of glutathione-ascorbate cycle that causes ROS damages. Thioredoxin 1 and thioredoxin reductase (NADPH) were down-regulated in JA-CMS as well (Table 4). Although not directly involved in glutathione-ascorbate cycle, thioredoxins are involved in oxidative damage avoidance by supplying reducing power to reductases detoxifying lipid hydroperoxides or repairing oxidized proteins [37]. Overall, increased production of ROS through photosynthesis plus decreased enzymatic activity of ROS-scavenging enzymes may dramatically perturb ROS homeostasis in JA-CMS.

**Table 4:** Down-regulated oxidoreductase and alpha-linolenic acid metabolism genes in JA-CMS at the SS and/or MS stages.

Category	Function	Gene ID	SS stage			MS stage		
			Fold	P value	Q value	Fold	P value	Q value
Oxidoreductase	Iron/ascorbate family oxidoreductase	comp74308_c0	−2.17	1.52E-19	4.38E-17	−2.46	1.15E-30	6.70E-28
		comp75973_c0	−1.29	1.58E-14	3.19E-12	−1.60	6.00E-28	2.92E-25
		comp76619_c0	−1.97	2.58E-69	4.05E-66	−1.58	3.31E-40	2.66E-37
		comp76821_c0	−1.55	6.12E-11	8.77E-09	−1.12	1.53E-06	1.34E-04
		comp77552_c0	−3.93	8.85E-91	2.22E-87	−2.97	1.16E-42	1.06E-39
		comp78972_c0	−4.08	8.28E-77	1.67E-73	−2.89	8.24E-43	7.64E-40
		comp79641_c0	−1.96	5.14E-05	3.22E-03	−2.35	6.26E-07	5.81E-05
		comp79893_c0	−1.16	1.52E-13	2.81E-11	−1.02	9.31E-12	1.54E-09
	Monodehydroascorbate reductase (MDHAR)	comp73498_c0	−4.21	1.43E-07	1.37E-05	Not significant		
	Glutathione peroxidase (GPX)	comp73626_c0	−1.45	2.69E-80	6.02E-77	−1.55	5.17E-97	1.58E-93
	Glutathione S-transferase (GST)	comp40493_c0	−2.71	1.54E-11	2.36E-09	−2.46	3.63E-13	6.88E-11
		comp74280_c0	−1.66	3.00E-14	5.83E-12	−1.32	1.37E-10	2.00E-08
		comp76601_c0	−1.85	3.65E-15	7.60E-13	−1.77	2.14E-16	5.29E-14
		comp81581_c0	−1.54	1.40E-05	1.01E-03	−1.36	4.44E-05	2.95E-03
		comp92611_c0	−2.96	1.61E-06	1.35E-04	−2.32	3.14E-06	2.60E-04
	Thioredoxin 1	comp63917_c0	Not significant			−1.57	4.86E-07	4.58E-05
	Thioredoxin reductase (NADPH)	comp87684_c1	−1.17	1.99E-12	3.35E-10	−1.20	1.21E-13	2.37E-11
Alpha-linolenic acid metabolism	Allene oxide synthase (AOS)	comp75493_c0	−1.45	1.04E-127	5.93E-124	−1.02	1.57E-58	2.34E-55
	Allene oxide cyclase (AOC)	comp70218_c0	−2.68	1.51E-10	2.09E-08	−2.03	1.63E-08	1.83E-06
		comp80154_c0	−1.75	4.08E-59	5.11E-56	−1.61	7.73E-53	9.09E-50
	OPDA (12-oxophytodienoic acid) reductase	comp79354_c1	−2.83	8.17E-85	1.97E-81	−2.84	1.73E-71	3.53E-68
	OPC-8:0 CoA ligase 1	comp82201_c0	−1.01	8.43E-29	3.95E-26	Not significant		
	Acyl-CoA oxidase (AOX)	comp86147_c0	−1.80	4.97E-105	1.64E-101	−1.63	4.80E-79	1.18E-75
	Acetyl-CoA acyltransferase 1 (ACAA)	comp80628_c2	-1.09	7.33E-17	1.73E-14	Not significant		
	Alpha-dioxygenase	comp66758_c0	−5.66	2.09E-11	3.15E-09			

Note: Fold = log_2_(fold change). Significant DE genes were determined based on |log_2_(fold change)|>1 and FDR q value<0.005.

The “alpha-linolenic acid metabolism” pathway was another one that was significantly over-represented in KEGG analysis of down-regulated DE genes at the SS stage in JA-CMS. Although this pathway was only detected for the SS stage, genes encoding key enzymes in this pathway were significantly down-regulated in JA-CMS at both SS and/or MS stages, which include allene oxide synthase gene (AOS), 2 allene oxide cyclase genes (AOC), OPDA (12-oxo-phytodienoic acid) reductase gene, OPC-8:0 (3-oxo-2((2Z)-pentenyl)-cyclopentane-1-octanoic acid) CoA ligase gene, acyl-CoA oxidase gene (AOX), acetyl-CoA acyltransferase gene (ACAA), and α-dioxygenase gene (Table 4). Among these genes, α-dioxygenase gene is worth noting, because its transcript abundance decreased around 50-fold in JA-CMS comparing with its counterpart at the SS stage; and it was reported to protect against oxidative stress and cell death in *Arabidopsis* [38], lack of which may exacerbate ROS imbalance as aforementioned.

The “alpha-linolenic acid metabolism” pathway belongs to jasmonic acid biosynthesis module according to KEGG pathway database. Multiple studies have presented the association between jasmonic acid biosynthesis and CMS. McConn and Browse [39] found that *Arabidopsis* triple mutants that contain negligible levels of trienoic fatty acids were male sterile, and exogenous applications of α-linoleate or jasmonate restored fertility. An *Arabidopsis* knock-out mutant defective in the AOS gene *CYP74A* also showed severe male sterility due to defects in anther and pollen development; this phenotype was completely rescued by exogenous application of methyl jasmonate and by complementation with constitutive expression of the AOS gene [40]. In addition, aberrant jasmonic acid pathway was detected in CMS rice during the development of microspores [41].

Besides photosynthesis related genes, the “flavonoid biosynthesis” pathway were over-represented with up-regulated DE genes in JA-CMS at the SS and MS stages as well. This pathway comprises 10 up-regulated genes in this study, which are 4 chalcone synthase genes, one of the key enzymes for flavonoid biosynthesis, 1 anthocyanidin reductase gene, 1 leucoanthocyanidin dioxygenase gene, 1 bifunctional dihydrofavonol 4-reductase/flavanone 4-reductase gene, 1 trans-cinnamate 4-monooxygenase gene, 1 flavonoid 3′, 5′-hydroxylase gene and 1 naringenin 3-dioxygenase gene (Table 3). However, two flavonoid biosynthesis related genes were found down-regulated in JA-CMS simultaneously; they are 1 chalcone synthase gene with a 4-fold expression decrease (q = 2.21×10^−3^) at the SS stage, and 1 caffeoyl-CoA O-methyltransferase gene down-regulated at both SS (2-fold, q = 1.05×10^−5^) and MS stages (2-fold, q = 3.44×10^−7^).

In literature, several nuclear genes pertaining to flavonoid biosynthesis were reported to be inhibited in Ogura CMS of *Raphanus sativus*. The expression of chalcone synthase gene was strongly inhibited in the later stages of anther development in sterile cytoplasm [42]. Wei et al. [43] implemented digital gene expression analysis to compare gene expressions of wild type and genetic male sterility (GMS) mutant cotton at the meiosis, tetrad, and uninucleate microspore stages, respectively. Genes involved in flavonoid metabolism, such as CHS, flavonoid 3′, 5′-hydroxylase, anthocyanidin reductase, and leucoanthocyanidin reductase were down-regulated at the meiosis and uninucleate microspore stages, but were up-regulated at the tetrad stage, in GMS mutant anthers. Differences between the current study and these findings may lie in diverse plant materials, different sterility mechanisms and distinct development stages. Nevertheless contradictory results, flavonoids have been suggested to constitute a secondary ROS-scavenging system in plants exposed to severe stress conditions [44] and genes in flavonoid biosynthesis are important in anther and pollen development. Further studies are warranted to disentangle specific functions of these genes in balancing ROS homeostasis at different development stages of sterile plants.

In addition, in GO analysis of DE genes between the two stages of the same material, ATP biosynthesis related categories were significantly enriched in sterile plants. Three out of the 9 down-regulated genes identified in this comparison were involved in ATP biosynthesis. The three DE genes, only detected in JA-CMS, are proton-transporting ATP synthase complex, coupling factor F_o_ (comp73972_c0; 3-fold expression decrease at the MS stage, q = 5.88×10^−85^), ATPase subunit 1 (comp84388_c5; 3-fold, q = 1.58×10^−5^), and ATP synthase CF1 alpha subunit (comp85965_c1; 2-fold, q = 4.19×10^−15^). Same expression patterns of ATP synthase related genes were observed in studies of Dong et al. [45] and Liu et al. [15].

ATP synthase, located within the plant mitochondria, consists of F_o_ and F_1_ regions, which plays an important role in providing energy. The F_o_ portion is within the membrane and functions as a proton pore; it consists of orf25, orfB, subunit 6 (*atp6*) and subunit 9 (*atp9*) [46], among which loci within or up/downstream *atp6* and *atp9* have been associated with CMS in a variety of plant species [47]–[50]. Comp73972_c0 in this study may be one of these genes. There have been at least 16 CMS-related ORFs containing structural genes, especially the ones encoding ATP-F_o_ and ATP-F_1_ [2], [51]–[54]. Cotton CMS studies have also demonstrated step by step that the *atp1* and *atp6* genes in CMS-D8 could be the candidates of CMS-associated genes in the mitochondrial genome [55], the abnormal sequence and expression of *atpA* gene is associated with CMS expression in Upland cotton [56], and PCR-based SNP markers in genes encoding ATP synthase subunits are useful in discriminating CMS-D8, CMS-D2 and Upland cotton cytoplasms [57]. Because of the increased energy demand during pollen development, one or a few gene product(s) that interferes with mitochondrial F_o_F_1_-ATP synthase function is likely to induce pollen sterility [58]. But based on the results presented here, dysfunctional ATP synthase may not be the major cause of CMS in this study.

In summary, as the first study using unbiased transcriptome sequencing technology to investigate CMS-associated genes in cotton, a substantial number of biologically meaningful DE genes, especially the ones related with photosynthesis, redox reactions, alpha-linolenic acid metabolism, and flavonoid biosynthesis, were identified. Profiling expression patterns of these genes across all stages of pollen development may provide additional evidence for their importance. Further experiments are warranted to elucidate molecular mechanisms of these genes that lead to CMS.

### Supplementary Note

Several CMS systems have been reported in cotton which include *Gossypiumar boreum*, *G. anomalum*, *G. harknessii*, *G. trilobum*, *G. barbadense* and *G. hirsutum*. CMS-D2 and CMS-D8 are the two most commonly studied systems among them.

The male sterile characteristic of JA-CMS is maintained by *G. hirsutum*, and its *restorer-of-fertility* (*Rf*) genes come from *G. barbadense*. The primitive type and natural outcrossing hybrids of JA-CMS resemble its female parent (*G. hirsutum*) [59]. However, the original *Rf* genes of CMS-D2 and 104-7A come from *G. harknessii* [17], [60]. And CMS-D8 is known to have its *Rf* genes come from its sister line [61].

Meanwhile, the mtDNA RFLP and PCR analysis results revealed that JA-CMS possesses a novel mitotype compared with the mitochondrial genome of either 104-7A or *G. harknessii* CMS systems [62], [63].

These evidence at genetic, morphological and molecular levels demonstrated that factors and mechanisms associated with CMS induction in JA-CMS may be different from CMS-D2, CMS-D8 and 104-7A.

## Supporting Information

Figure S1 Results of qRT-PCR. Columns and bars represented the means and standard errors (n = 3), respectively. B2 = SS stage of JA-CMS, K2 = SS stage of JB, B3 = MS stage of JA-CMS, K3 = MS stage of JB.
(TIF)

Figure S2 Transcript length distribution. Minimum, N90, mean, N50, and maximum lengths of transcripts are marked on the figure. The N90/N50 value is a weighted median and defined as the length of the smallest transcript S in the sorted list of all transcripts where the cumulative length from the largest transcript to S is at least 90%/50% of the total length. There are 206,496 transcripts in total; most of the transcripts (74,998 [36.3%]) have lengths between 200–500 base pairs.
(TIF)

Figure S3 Unigene length distribution. Minimum, N90, mean, N50, and maximum lengths of unigenes are marked on the figure. The N90/N50 value is a weighted median and defined as the length of the smallest unigene S in the sorted list of all unigenes where the cumulative length from the largest unigene to S is at least 90%/50% of the total length. A total of 86,093 unigenes were identified; 59.8% (51,491) of the unigenes have lengths between 200–500 base pairs.
(TIF)

Figure S4 RPKM density distributions of 854 DE genes. B2 = SS stage of JA-CMS, K2 = SS stage of JB, B3 = MS stage of JA-CMS, K3 = MS stage of JB.
(TIF)

Figure S5 GO analysis results of up-regulated DE genes in JA-CMS at the SS stage comparing with JB. BP = Biological process, CC = Cellular component, MF = Molecular function; B2 = SS stage of JA-CMS, K2 = SS stage of JB.
(TIF)

Figure S6 GO analysis results of down-regulated DE genes in JA-CMS at the SS stage comparing with JB. BP = Biological process, CC = Cellular component, MF = Molecular function; B2 = SS stage of JA-CMS, K2 = SS stage of JB.
(TIF)

Figure S7 GO analysis results of up-regulated DE genes in JA-CMS at the MS stage comparing with JB. BP = Biological process, CC = Cellular component, MF = Molecular function; B3 = MS stage of JA-CMS, K3 = MS stage of JB.
(TIF)

Figure S8 GO analysis results of down-regulated DE genes in JA-CMS at the MS stage comparing with JB. BP = Biological process, CC = Cellular component, MF = Molecular function; B3 = MS stage of JA-CMS, K3 = MS stage of JB.
(TIF)

Figure S9 GO analysis results of down-regulated DE genes at the MS stage comparing with the SS stage in JA-CMS. BP = Biological process, CC = Cellular component, MF = Molecular function; B3 = MS stage of JA-CMS, B2 = SS stage of JA-CMS.
(TIF)

Figure S10 KEGG analysis results of up-regulated DE genes in JA-CMS at the SS stage comparing with JB. Rich factor is the ratio between counts of DE genes and all annotated genes enriched in a certain pathway; qvalue is P value after multiple hypothesis testing correction with a range between 0 and 1. Twenty most significant pathways were plotted, when more than 20 pathways were identified.
(TIF)

Figure S11 KEGG analysis results of down-regulated DE genes in JA-CMS at the SS stage comparing with JB. Rich factor is the ratio between counts of DE genes and all annotated genes enriched in a certain pathway; qvalue is P value after multiple hypothesis testing correction with a range between 0 and 1. Twenty most significant pathways were plotted, when more than 20 pathways were identified.
(TIF)

Figure S12 KEGG analysis results of up-regulated DE genes in JA-CMS at the MS stage comparing with JB. Rich factor is the ratio between counts of DE genes and all annotated genes enriched in a certain pathway; qvalue is P value after multiple hypothesis testing correction with a range between 0 and 1. Twenty most significant pathways were plotted, when more than 20 pathways were identified.
(TIF)

Figure S13 KEGG analysis results of down-regulated DE genes in JA-CMS at the MS stage comparing with JB. Rich factor is the ratio between counts of DE genes and all annotated genes enriched in a certain pathway; qvalue is P value after multiple hypothesis testing correction with a range between 0 and 1. Twenty most significant pathways were plotted, when more than 20 pathways were identified.
(TIF)

Table S1 Gene-specific primers for qRT-PCR.
(DOCX)

Table S2 DE genes between JA-CMS and JB plants at the SS stage.
(XLSX)

Table S3 DE genes between JA-CMS and JB plants at the MS stage.
(XLSX)

Table S4 DE genes between the SS and MS stages in JA-CMS.
(XLSX)

Table S5 DE genes between the SS and MS stages in JB.
(XLSX)
