# Supplementary figures and images for: Transcriptome Sequencing and De Novo Analysis of Cytoplasmic Male Sterility and Maintenance in JA-CMS Cotton
Source: PLoS One. 2014 Nov 5;9(11):e112320. doi: 10.1371/journal.pone.0112320 (PMC4221291; doi:10.1371/journal.pone.0112320)

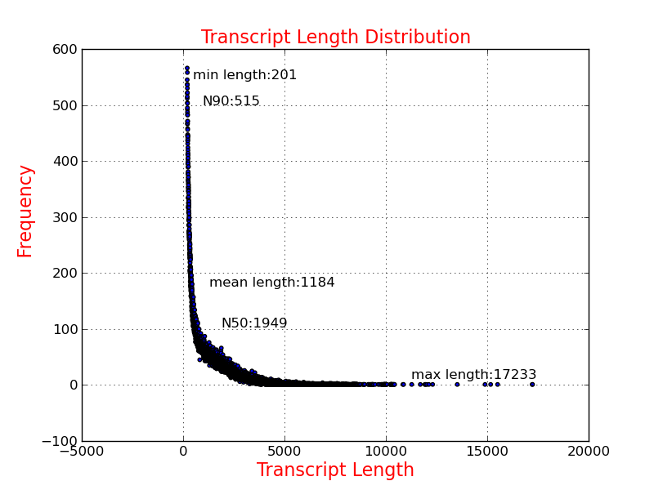

Supplement: Figure S2 — Transcript length distribution. Minimum, N90, mean, N50, and maximum lengths of transcripts are marked on the figure. The N90/N50 value is a weighted median and defined as the length of the smallest transcript S in the sorted list of all transcripts where the cumulative length from the largest transcript to S is at least 90%/50% of the total length. There are 206,496 transcripts in total; most of the transcripts (74,998 [36.3%]) have lengths between 200–500 base pairs. (TIF) [file pone.0112320.s002.tif]

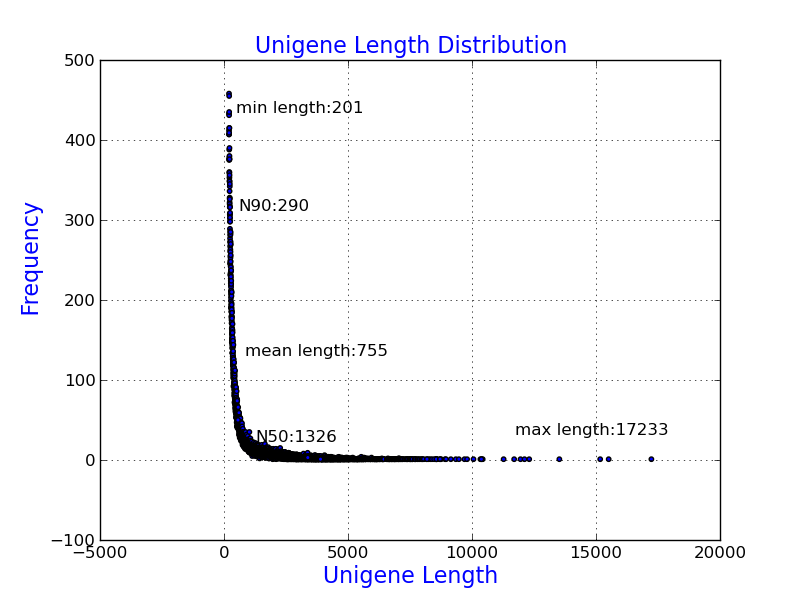

Supplement: Figure S3 — Unigene length distribution. Minimum, N90, mean, N50, and maximum lengths of unigenes are marked on the figure. The N90/N50 value is a weighted median and defined as the length of the smallest unigene S in the sorted list of all unigenes where the cumulative length from the largest unigene to S is at least 90%/50% of the total length. A total of 86,093 unigenes were identified; 59.8% (51,491) of the unigenes have lengths between 200–500 base pairs. (TIF) [file pone.0112320.s003.tif]

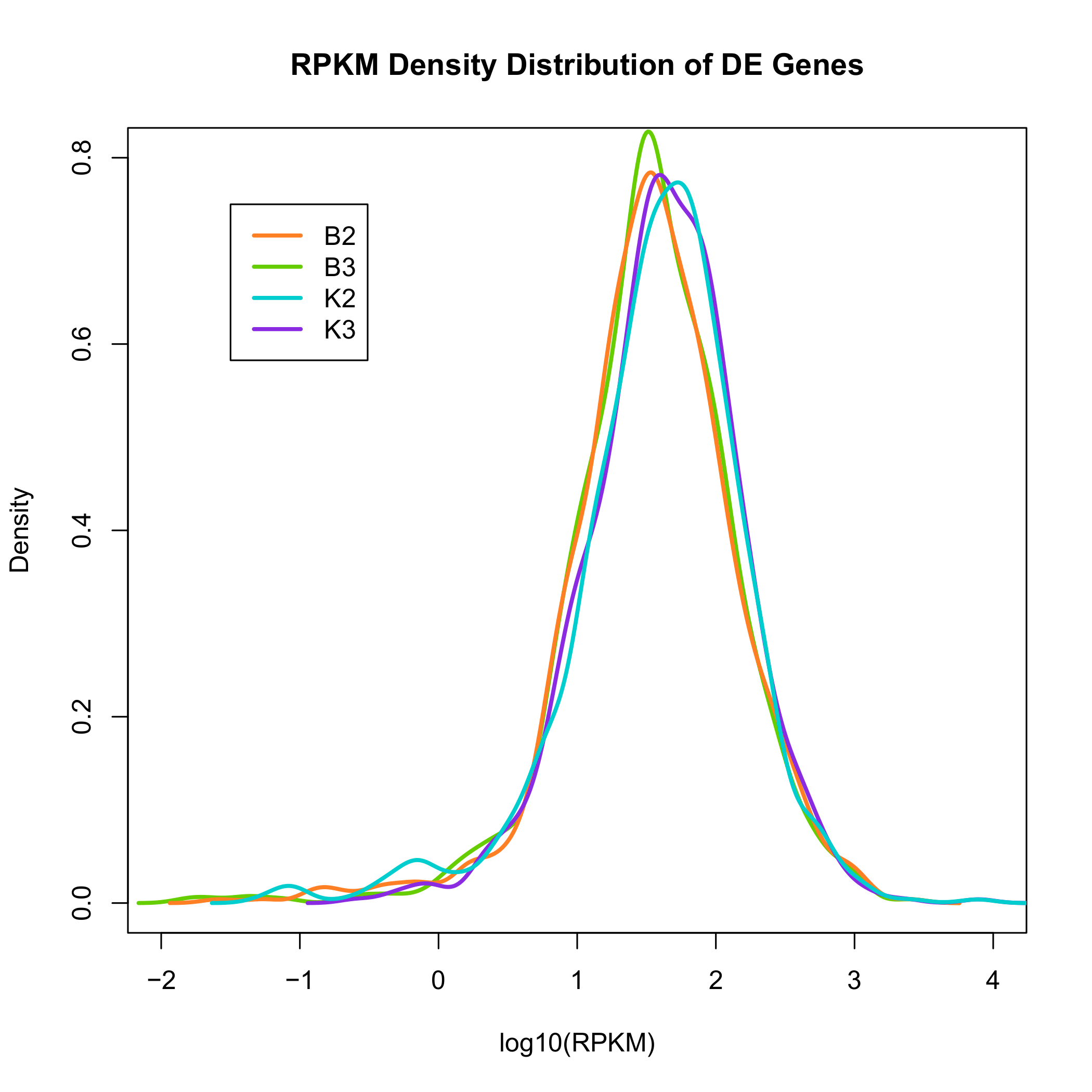

Supplement: Figure S4 — RPKM density distributions of 854 DE genes. B2 = SS stage of JA-CMS, K2 = SS stage of JB, B3 = MS stage of JA-CMS, K3 = MS stage of JB. (TIF) [file pone.0112320.s004.tif]

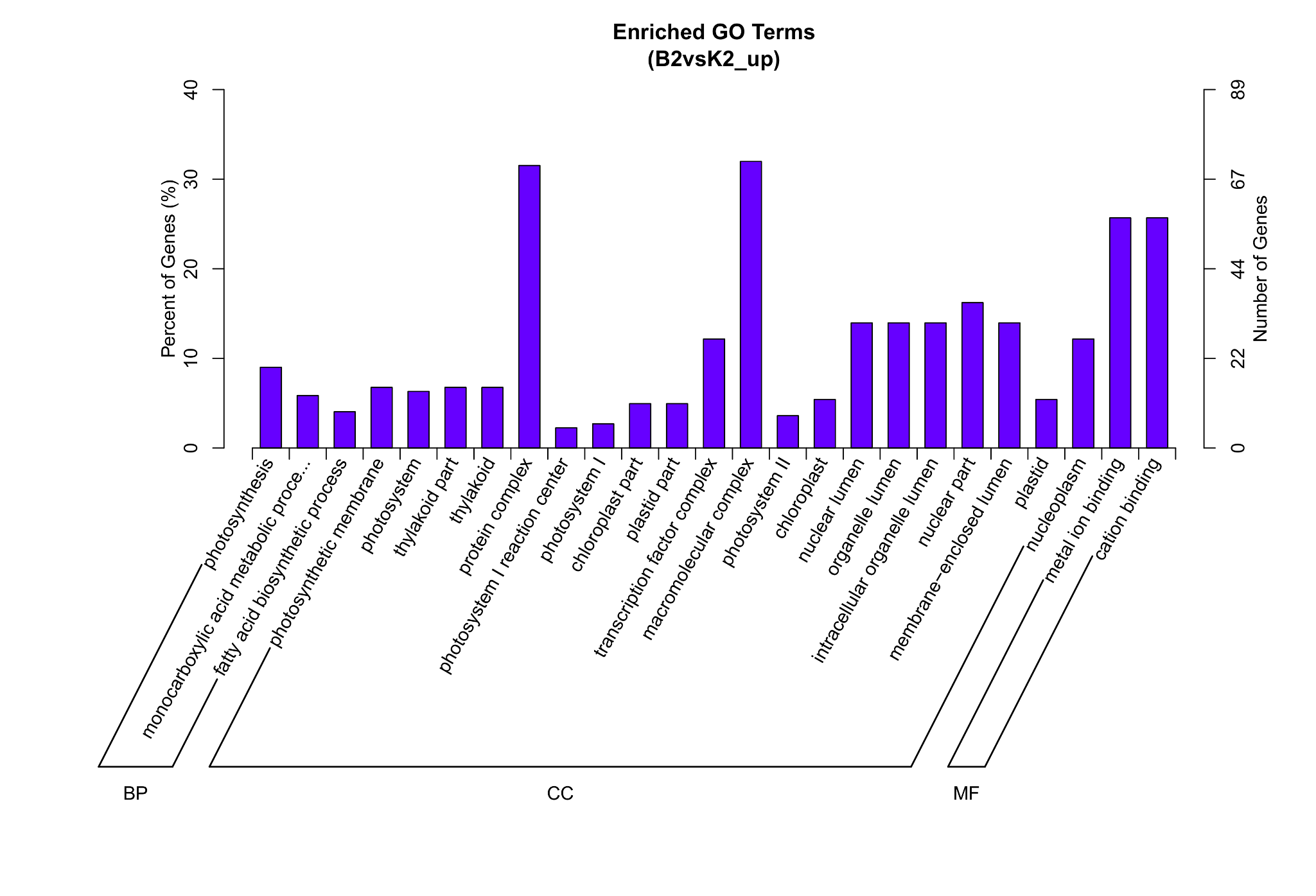

Supplement: Figure S5 — GO analysis results of up-regulated DE genes in JA-CMS at the SS stage comparing with JB. BP = Biological process, CC = Cellular component, MF = Molecular function; B2 = SS stage of JA-CMS, K2 = SS stage of JB. (TIF) [file pone.0112320.s005.tif]

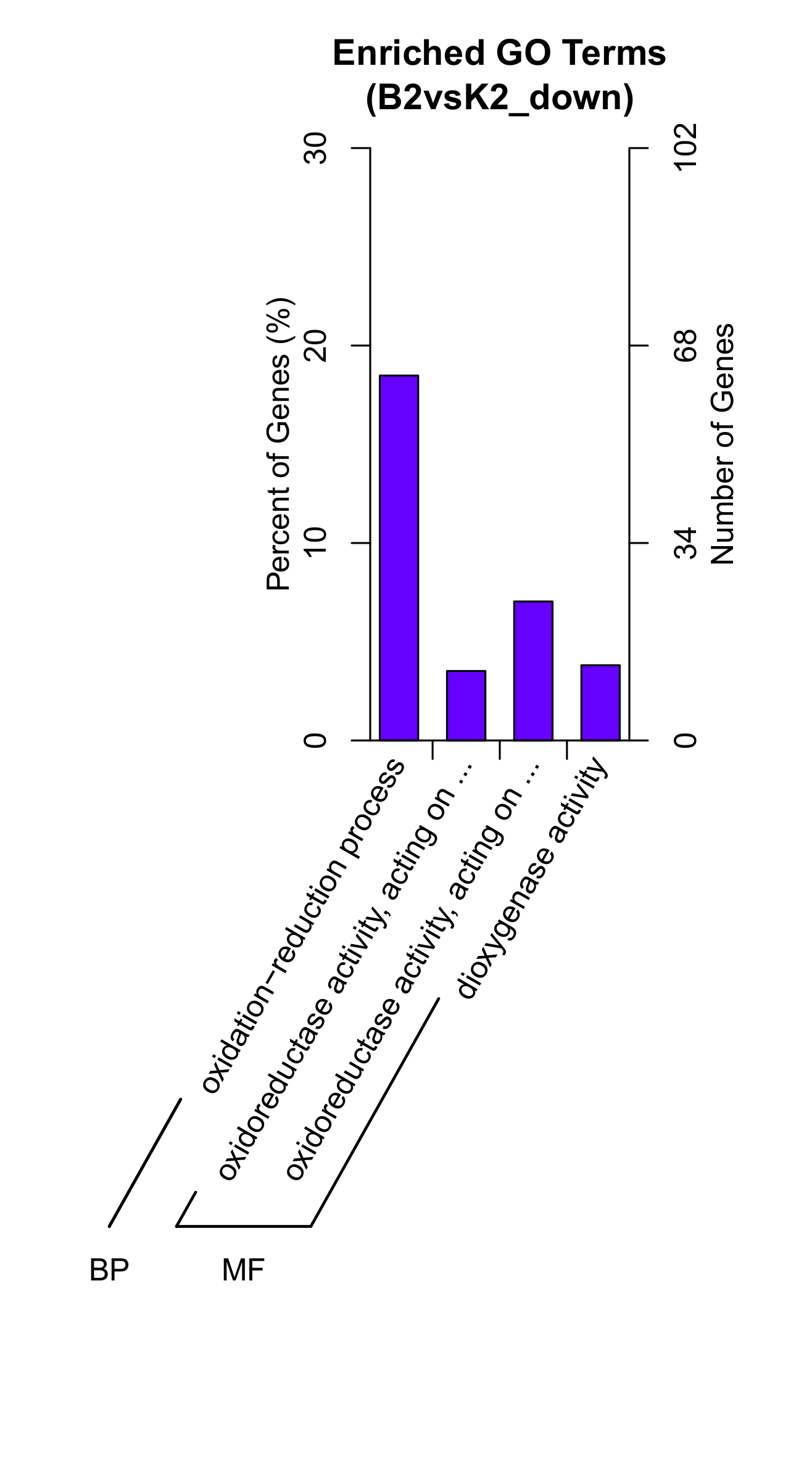

Supplement: Figure S6 — GO analysis results of down-regulated DE genes in JA-CMS at the SS stage comparing with JB. BP = Biological process, CC = Cellular component, MF = Molecular function; B2 = SS stage of JA-CMS, K2 = SS stage of JB. (TIF) [file pone.0112320.s006.tif]

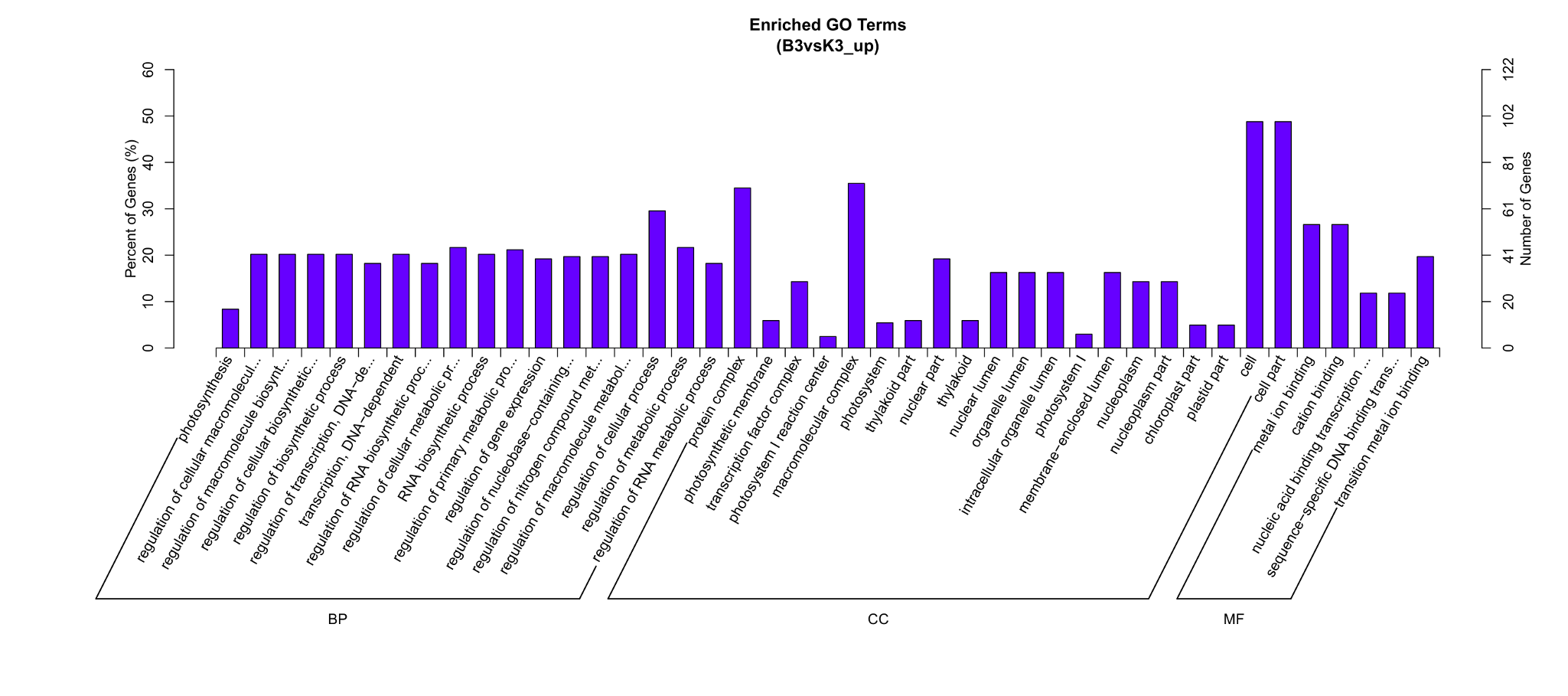

Supplement: Figure S7 — GO analysis results of up-regulated DE genes in JA-CMS at the MS stage comparing with JB. BP = Biological process, CC = Cellular component, MF = Molecular function; B3 = MS stage of JA-CMS, K3 = MS stage of JB. (TIF) [file pone.0112320.s007.tif]

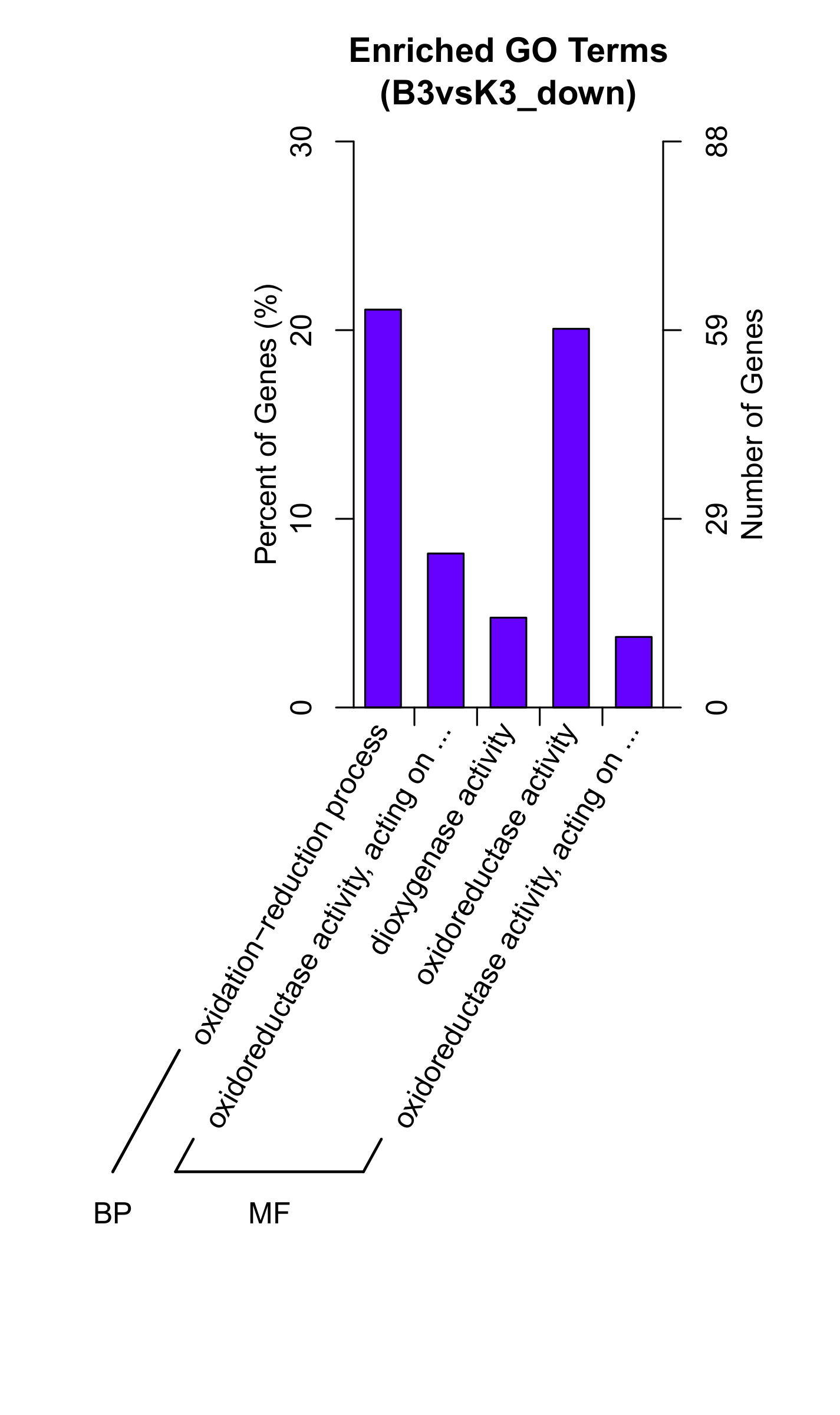

Supplement: Figure S8 — GO analysis results of down-regulated DE genes in JA-CMS at the MS stage comparing with JB. BP = Biological process, CC = Cellular component, MF = Molecular function; B3 = MS stage of JA-CMS, K3 = MS stage of JB. (TIF) [file pone.0112320.s008.tif]

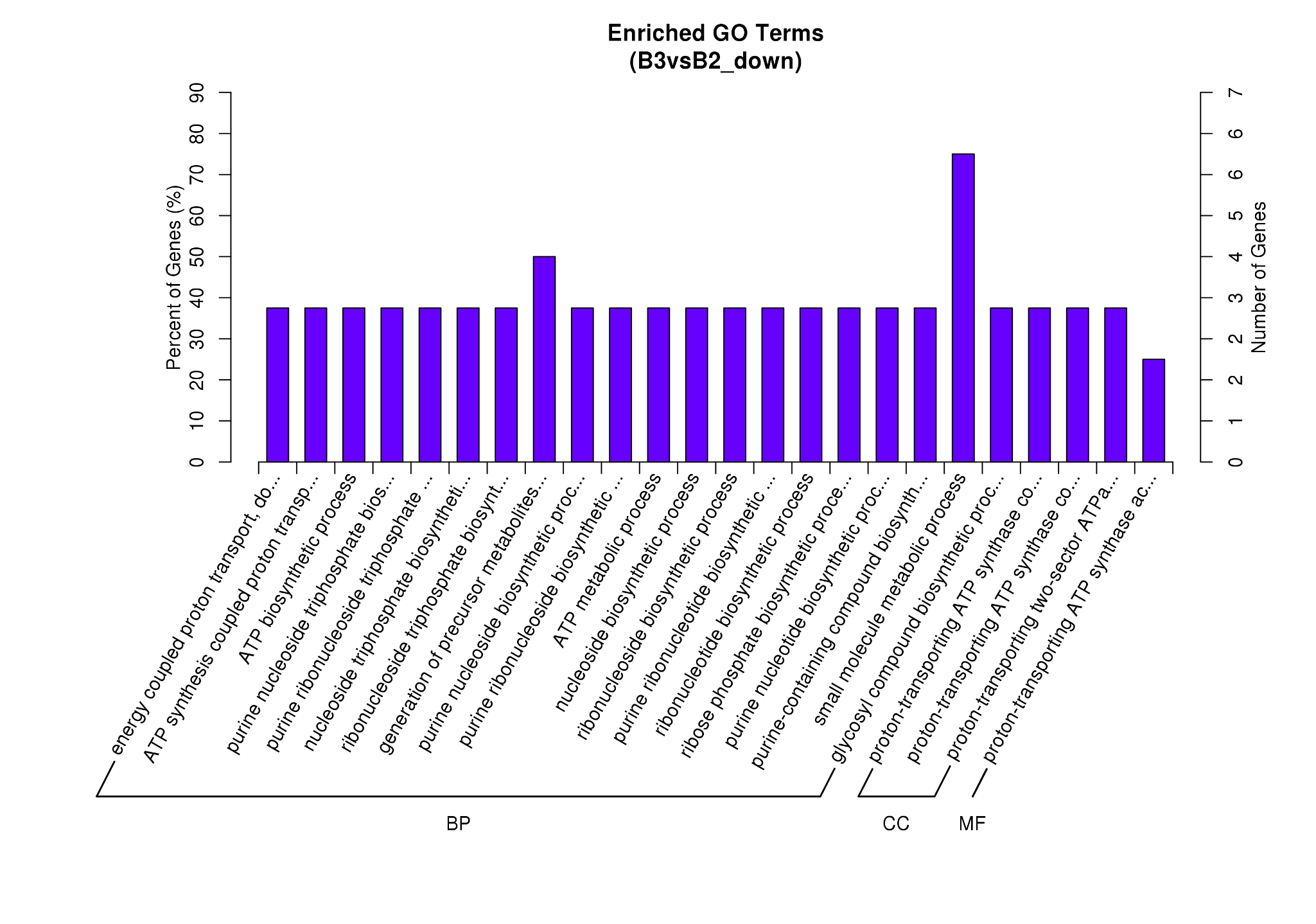

Supplement: Figure S9 — GO analysis results of down-regulated DE genes at the MS stage comparing with the SS stage in JA-CMS. BP = Biological process, CC = Cellular component, MF = Molecular function; B3 = MS stage of JA-CMS, B2 = SS stage of JA-CMS. (TIF) [file pone.0112320.s009.tif]

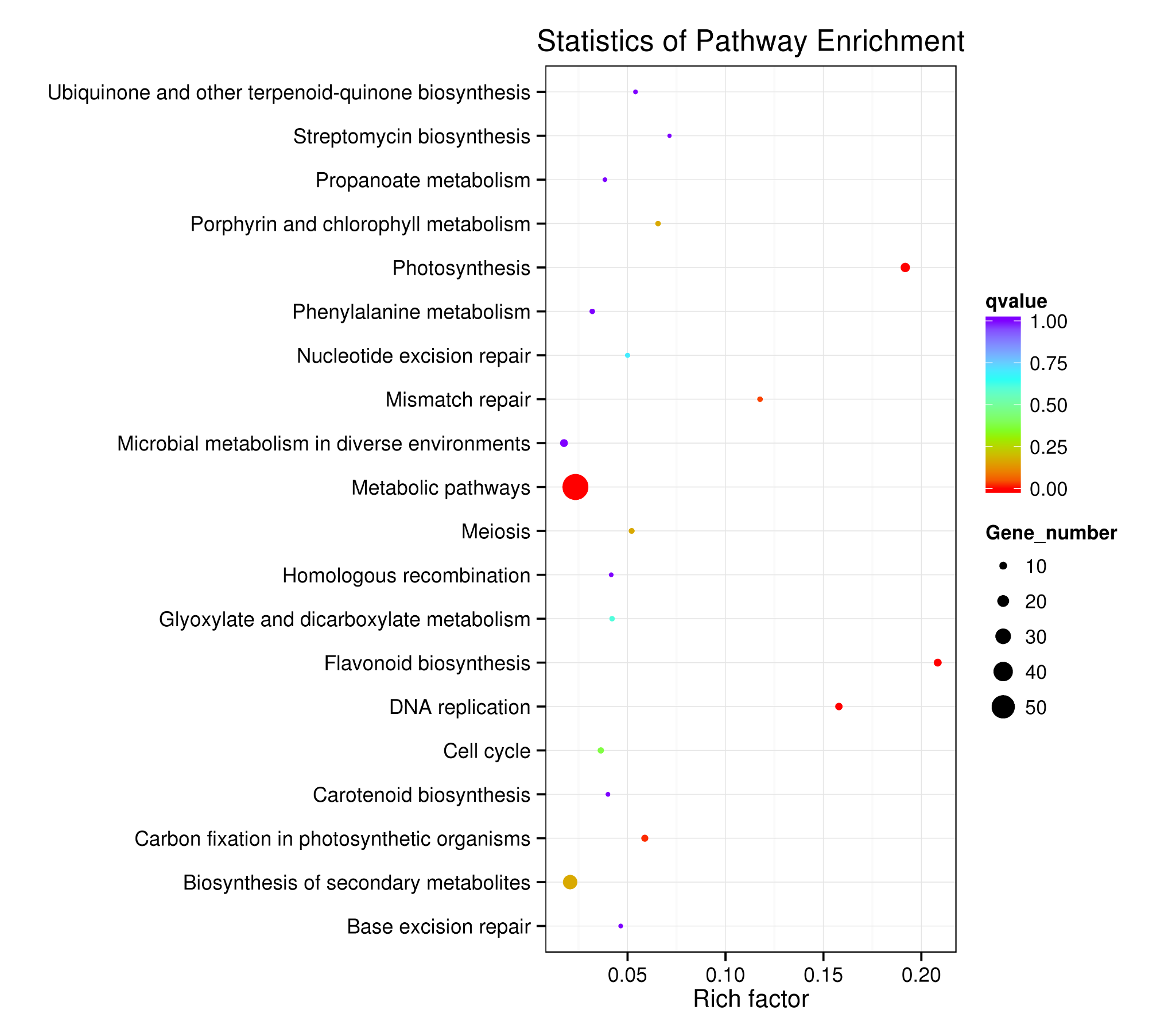

Supplement: Figure S10 — KEGG analysis results of up-regulated DE genes in JA-CMS at the SS stage comparing with JB. Rich factor is the ratio between counts of DE genes and all annotated genes enriched in a certain pathway; qvalue is P value after multiple hypothesis testing correction with a range between 0 and 1. Twenty most significant pathways were plotted, when more than 20 pathways were identified. (TIF) [file pone.0112320.s010.tif]

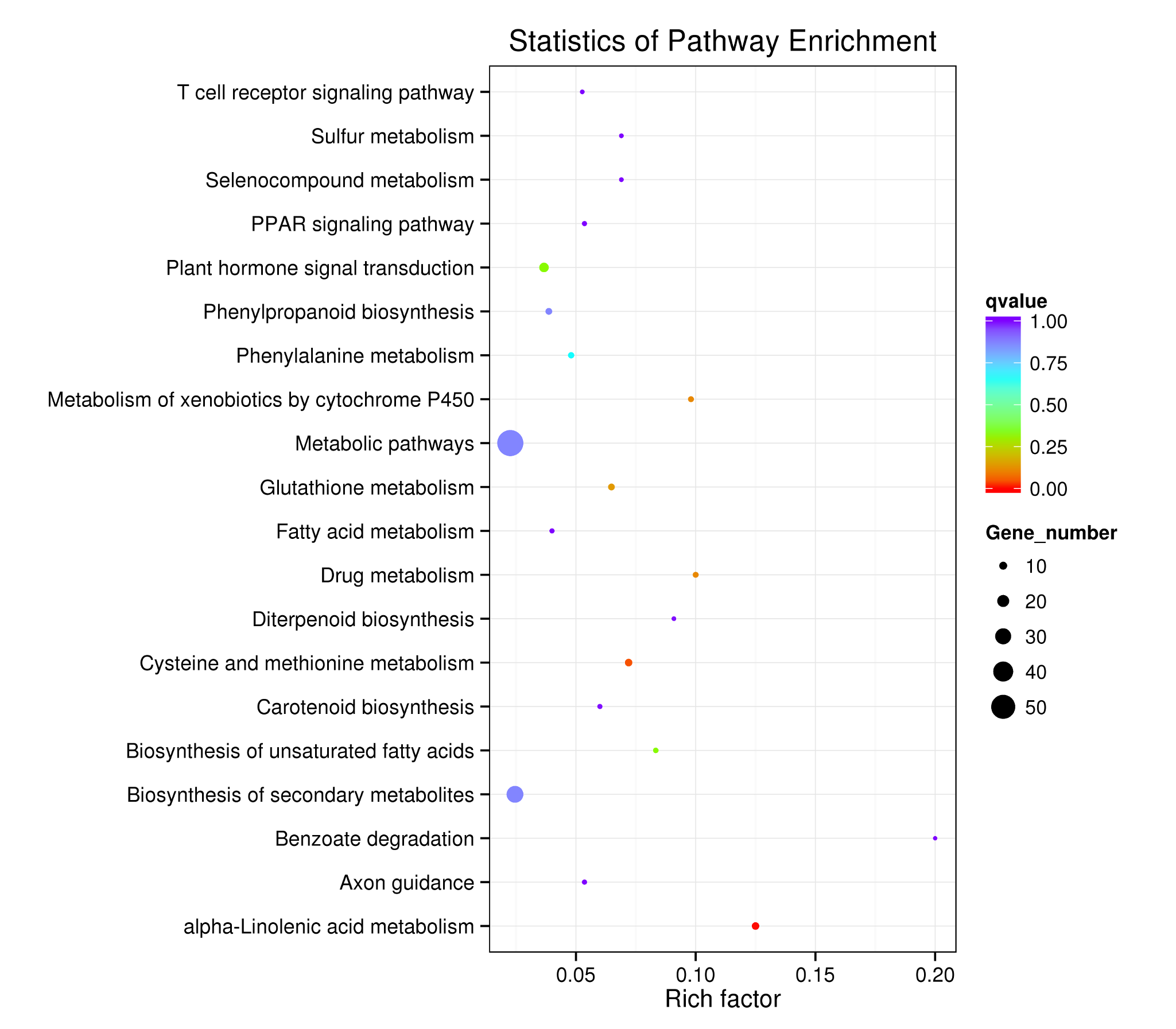

Supplement: Figure S11 — KEGG analysis results of down-regulated DE genes in JA-CMS at the SS stage comparing with JB. Rich factor is the ratio between counts of DE genes and all annotated genes enriched in a certain pathway; qvalue is P value after multiple hypothesis testing correction with a range between 0 and 1. Twenty most significant pathways were plotted, when more than 20 pathways were identified. (TIF) [file pone.0112320.s011.tif]

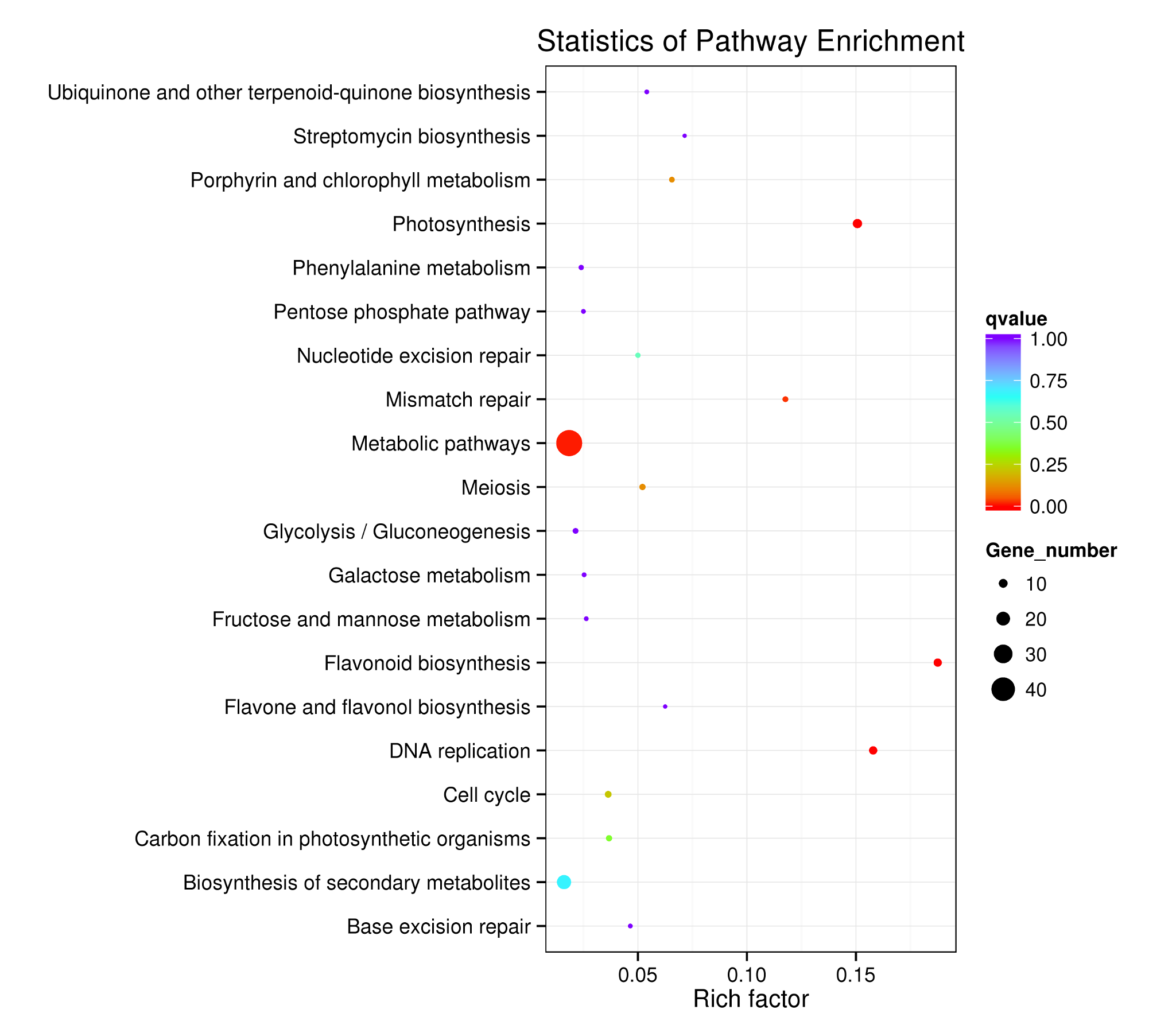

Supplement: Figure S12 — KEGG analysis results of up-regulated DE genes in JA-CMS at the MS stage comparing with JB. Rich factor is the ratio between counts of DE genes and all annotated genes enriched in a certain pathway; qvalue is P value after multiple hypothesis testing correction with a range between 0 and 1. Twenty most significant pathways were plotted, when more than 20 pathways were identified. (TIF) [file pone.0112320.s012.tif]

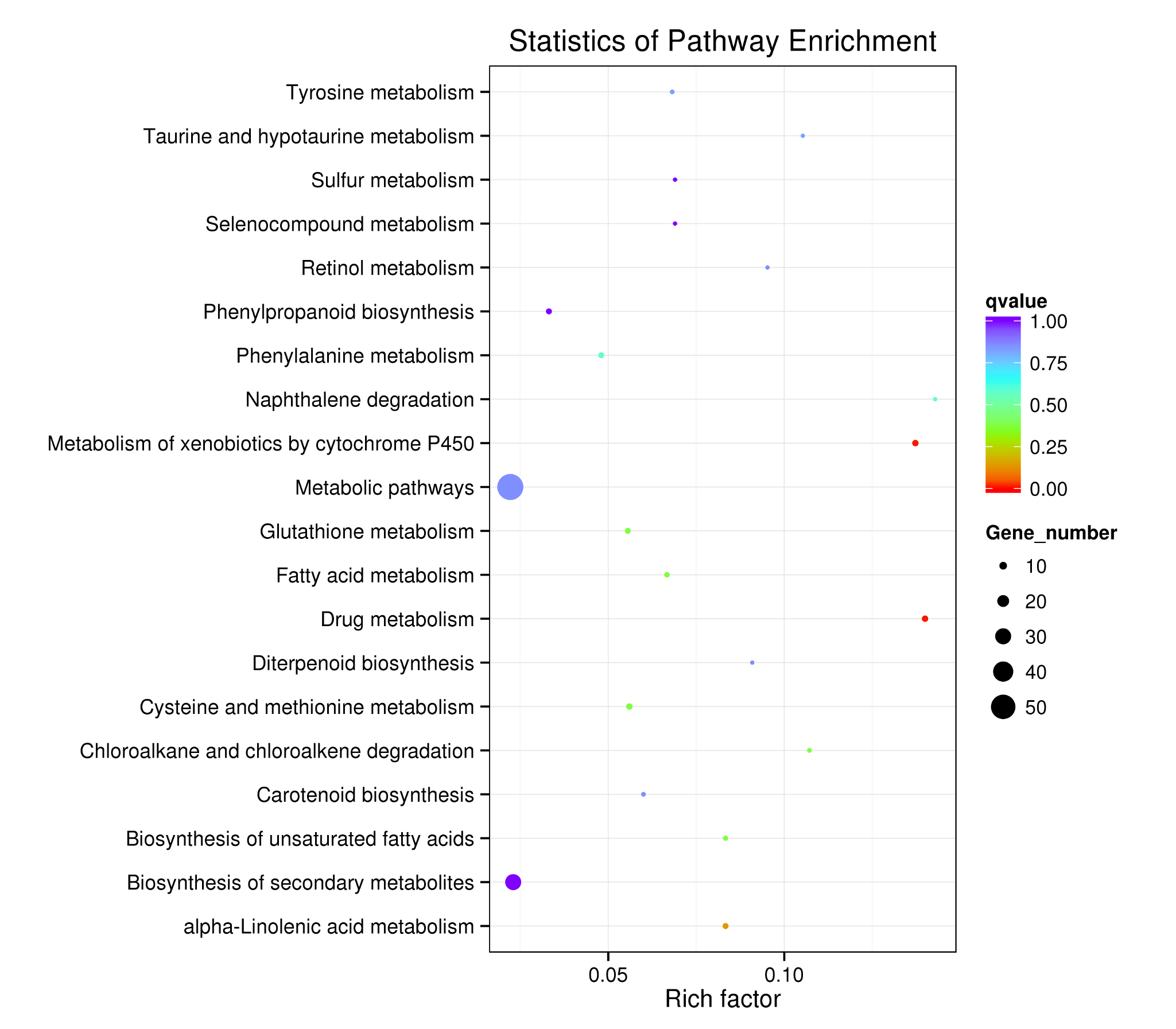

Supplement: Figure S13 — KEGG analysis results of down-regulated DE genes in JA-CMS at the MS stage comparing with JB. Rich factor is the ratio between counts of DE genes and all annotated genes enriched in a certain pathway; qvalue is P value after multiple hypothesis testing correction with a range between 0 and 1. Twenty most significant pathways were plotted, when more than 20 pathways were identified. (TIF) [file pone.0112320.s013.tif]
