# Supplementary material for: Transcriptome Sequencing and De Novo Analysis of Cytoplasmic Male Sterility and Maintenance in JA-CMS Cotton
Source: PLoS One. 2014 Nov 5;9(11):e112320. doi: 10.1371/journal.pone.0112320 (PMC4221291; doi:10.1371/journal.pone.0112320)
Supplement: Table S1 — Gene-specific primers for qRT-PCR. (DOCX) [file pone.0112320.s014.docx]

**Table S1.** Gene-specific primers for qRT-PCR.

| **Gene ID** | **Function description** | **Primer sequences** | **GC (%)** | **Length (bp)** | **Tm (°C)** |
| --- | --- | --- | --- | --- | --- |
| comp54402_c0 | 1-aminocyclopropane-1-carboxylate synthase | F:ATCTATGCTGCCACTGTCTTTG | 45.45 | 22 | 58.2 |
|  |  | R:AATGAGTACACTATCCCGACCC | 50.00 | 22 | 60.1 |
| comp77990_c0 | Alpha-expansin 11 precursor, putative [*Ricinus communis*] | F:TGTGGGTATGGGAACTTGTATTC | 43.48 | 23 | 57.8 |
|  |  | R:TGCTGTAATGGTCACGGATGT | 47.62 | 21 | 58.2 |
| comp71873_c0 | Predicted protein [*Populus trichocarpa*] | F:AAAAGACCCAAACTATCCACAAC | 39.13 | 23 | 56.8 |
|  |  | R:AATAAACAAACCAGGGCAACAC | 40.91 | 22 | 56.3 |
| comp68006_c0 | Conserved hypothetical protein [*Ricinus communis*] | F:CTCTTGCCGCCGCTGATT | 61.11 | 18 | 56.6 |
|  |  | R:GTTTCTGCCATTGTCGTTCTC | 47.62 | 21 | 58.0 |
| comp70789_c0 | Chloroplast chlorophyll A-B binding protein [*Gossypium hirsutum*] | F:GCTGATCCCGAGACCTTTGC | 60.00 | 20 | 61.9 |
|  |  | R:CCCGGCCTTGAACCATACA | 57.89 | 19 | 58.7 |
| comp88938_c1 | Transcription factor LHY [*Populus nigra*] | F:TTCAAGATTCCGTATTTCACCC | 40.91 | 22 | 56.3 |
|  |  | R:AAACCTCTTAGTTCATTTGTGGG | 39.13 | 23 | 56.6 |
| comp86790_c1 | *G. hirsutum* cab gene for chlorophyll A-B binding protein | F:GCCCTGACCGCCCAAAGTA | 63.16 | 19 | 60.1 |
|  |  | R:GCACCAAGCATTGCCCATC | 57.89 | 19 | 60.0 |
| EF-1α | Elongation factor 1α | F:GCGATCTGGTAAGGAGCTTG | 55.00 | 20 | 60.0 |
|  |  | R:GGAGAAGGTTTCCACAACCA | 50.00 | 20 | 59.0 |

Notes: F = Forward primer sequence; R = Reverse primer sequence; bp = Base pair; Tm = Melting temperature of the primer.
